# Extracellular vesicles in vascular cognitive impairment and dementia: a neurovascular evidence map for patient stratification and clinical translation

**DOI:** 10.3389/fneur.2026.1986081

**Published:** 2026-10-30

**Authors:** Siqi Li, Junli Liu

**Affiliations:** 1 College of Clinical Medicine, Qinghai University, Xining, Qinghai, China; 2 Department of Geriatrics, Affiliated Hospital of Qinghai University, Xining, Qinghai, China

**Keywords:** biomarkers, clinical translation, extracellular vesicles, neurovascular unit, patient stratification, vascular cognitive impairment and dementia

## Abstract

Vascular cognitive impairment and dementia (VCID) comprises distinct syndromes caused by cerebrovascular injury and mixed vascular-neurodegenerative pathology. Extracellular vesicles (EVs), including small EV-enriched preparations, are being investigated as biomarkers and therapeutic products. We performed a structured narrative review and an expanded PubMed search through 8 October 2026, incorporating exosome, microvesicle and microparticle terminology. The revision search retrieved 140 records; representative evidence tables summarize 13 preclinical therapeutic studies and 10 human biomarker studies. We organize the evidence by EV preparation, neurovascular unit mechanism, clinical phenotype and development pathway. Therapeutic evidence remains predominantly preclinical. Human studies include exploratory cohorts and studies with separate validation datasets or longitudinal follow-up, but prospective clinical utility remains unestablished. Differences in EV separation, characterization, purity and cellular attribution limit cross-study comparison. We propose five evidence domains covering biological rationale, technical control, phenotype-relevant translation, clinical validation and prospective evaluation. This author-developed map integrates existing EV, biomarker and VCID development principles. It has not undergone consensus development, inter-rater reliability testing or prospective validation, and does not assign readiness scores or mandate sequential progression. Its intended use is to make evidence gaps explicit and connect candidate assays or products to defined patient populations, outcomes and safety requirements.

## 1 Introduction

Vascular cognitive impairment and dementia (VCID) is a spectrum of cognitive syndromes caused by cerebrovascular injury and by interactions between vascular and neurodegenerative pathology. It includes post-stroke cognitive impairment, cerebral small vessel disease (CSVD)-related cognitive decline, chronic hypoperfusion-associated white matter injury, mixed Alzheimer disease (AD) and vascular pathology, and advanced vascular dementia (VaD). These phenotypes differ in lesion burden, blood–brain barrier (BBB) injury, inflammatory activity, white matter integrity, cognitive domains and disease stage. A single biomarker or therapeutic strategy is therefore unlikely to represent the entire spectrum (1–6).

Extracellular vesicles (EVs) provide a view of communication among endothelial cells, pericytes, immune cells, glia and neurons. EV-enriched preparations carry miRNAs, proteins, lipids and metabolites, and experimental studies link these components to inflammation, BBB stability, mitochondrial stress, myelin injury, neurogenesis and synaptic plasticity (7–19). Small EVs (sEVs) form an operational size category, commonly below 200 nm, rather than a uniform biological product. Reported EV effects establish biological plausibility but do not identify which patients would benefit or establish clinical utility.

Recent reviews synthesize EV mechanisms, diagnostic applications and stem cell-derived therapies in vascular dementia (20–24). Minimal Information for Studies of Extracellular Vesicles 2023 (MISEV2023), biomarker-development literature and VCID guidance already describe essential requirements for characterization and clinical translation (1, 5, 6, 25). Our contribution is an integrative neurovascular unit (NVU) evidence map that relates those requirements to defined VCID phenotypes and keeps biomarker development separate from therapeutic-product development. We connect reported EV source, cargo and recipient cell with imaging, cognition, assay performance, product attributes and safety. The five evidence domains are an author-proposed organizing tool whose reliability and predictive value remain untested (Figure 1).

**Figure 1:**
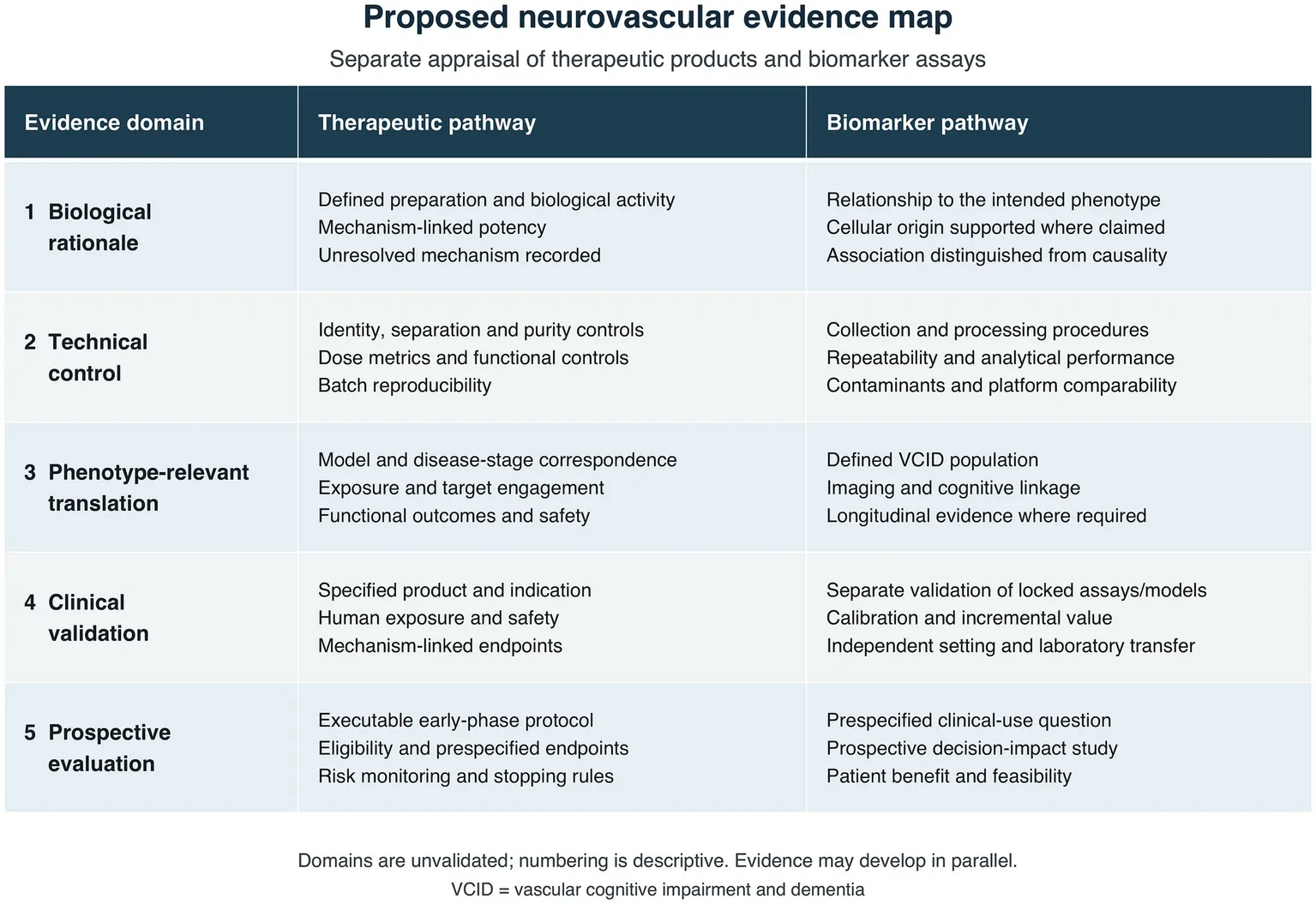
Five proposed evidence domains for neurovascular evidence mapping. The domains organize biological rationale, technical control, phenotype-relevant translation, clinical validation and prospective evaluation in separate biomarker and therapeutic pathways. Numbering identifies domains rather than validated readiness levels. Evidence may develop in parallel. The map integrates existing development principles and has not undergone consensus, inter-rater reliability testing or prospective validation.

## 2 Scope, search strategy and evidence selection

### 2.1 Structured narrative search

This is a structured narrative review, not a systematic review or meta-analysis. The original submission reported a PubMed search through 27 July 2026 and a 70-record core set. The original search export and screening log were not retained, so those counts and the original selection process cannot be independently reconstructed. For this revision, an expanded PubMed search was executed on 8 October 2026 from database inception using a publication-date upper limit of that date. The EV block includes extracellular vesicles, exosomes, microvesicles, microparticles, ectosomes, small EVs and extracellular particles. The disease block includes VCID, vascular dementia, small-vessel disease, cerebral hypoperfusion, post-stroke cognitive impairment and ischemic or hypoperfused white matter. Table 1 gives the executable strategy; Supplementary material S1 provides the returned records and narrative disposition notes. Backward citation tracing and targeted source checks supplement PubMed for contextual methodology and development guidance (1, 6, 20–23, 25). This process does not establish exhaustive coverage across databases.

**Table 1:** Expanded revision-stage PubMed search.

Search component	Executable PubMed text	Yield
EV block	(“Extracellular Vesicles”[MeSH] OR “extracellular vesicle*”[tiab] OR exosome*[tiab] OR microvesicle*[tiab] OR microparticle*[tiab] OR ectosome*[tiab] OR “small EV*”[tiab] OR “extracellular particle*”[tiab])	Not a separate search
Disease block	(“Dementia, Vascular”[MeSH] OR “vascular cognitive impairment”[tiab] OR “vascular cognitive impairment and dementia”[tiab] OR “vascular dementia”[tiab] OR “cerebral small vessel disease”[tiab] OR “small vessel disease”[tiab] OR “chronic cerebral hypoperfusion”[tiab] OR “cerebral hypoperfusion”[tiab] OR “post-stroke cognitive impairment”[tiab] OR “poststroke cognitive impairment”[tiab] OR (“white matter”[tiab] AND (ischemi*[tiab] OR ischaemi*[tiab] OR hypoperfus*[tiab])))	Not a separate search
Combined search, 8 October 2026	(EV block) AND (Disease block) AND (“1900/01/01”[Date - Publication]: “2026/10/08”[Date - Publication])	140 records

Search yield is a new revision-stage retrieval count. The original July 2026 record is unavailable. The complete expanded query and PMID list are archived in Supplementary material S1.

The expanded query retrieved 140 records. This is a new revision-stage search, not a reconstruction of the original 70-record set, and the difference cannot be interpreted as the number of newly eligible studies. Search yields describe retrieval, not the number of studies supporting a clinical claim.

### 2.2 Inclusion, exclusion and screening record

Eligible primary evidence concerns EVs in VCID, vascular dementia, CSVD with imaging or cognitive outcomes, chronic cerebral hypoperfusion, or post-stroke cognitive impairment. Adult white matter or NVU studies supply mechanistic context when they report a directly relevant vascular or functional outcome. We distinguish direct VCID evidence from acute-stroke assay studies, asymptomatic CSVD, hereditary CSVD and mixed-pathology models. Reviews and methodological standards supply context rather than primary efficacy evidence (Table 2). Neonatal or perinatal injury, unrelated organ disease, non-EV circulating analytes and nonvascular neurodegeneration without a relevant vascular comparison are outside the primary evidence map.

**Table 2:** Scope and contribution of related reviews and guidance.

Source	Scope	Established contribution	Relation to this review
Disease-focused reviews (20–23)	EV mechanisms and therapies in VaD/VCID	Source categories, molecular pathways and therapeutic considerations	Provide biological synthesis that this evidence map builds on
Wu and Kong (24)	EV mechanisms, diagnosis and therapy in VCI	Integration of diagnostic and treatment applications	Shows that integrating biomarker and therapeutic topics is already part of the literature
MISEV2023 (25)	EV research methods and reporting	Terminology, separation, characterization, function and attribution	Basis for technical appraisal, not a VCID readiness scale
VCID biomarker and trial guidance (1, 5, 6)	Clinical phenotypes, imaging, validation and trial preparation	Biological and clinical validation and phenotype-relevant design	Basis for clinical domains and outcome selection
Present review	Neurovascular evidence mapping across VCID phenotypes	Qualitative links among preparation, phenotype, assay/product requirements and evidence gaps	Integrative synthesis; five-domain grouping remains author-proposed and unvalidated

Revision-stage title and abstract triage identified records relevant to the clinical, mechanistic and methodological scope. Representative studies were selected for detailed discussion to cover different VCID phenotypes, EV sources, separation methods and stages of biomarker or product development. Tables 3–5 summarize 13 preclinical therapeutic studies and 10 human biomarker studies; contextual studies are discussed separately. The search record distinguishes tabled studies, contextual candidates, out-of-scope records and correction notices. Selection and appraisal are qualitative and were assisted by AI-supported source extraction. They do not constitute independent duplicate screening or a formal risk-of-bias assessment. The original full-text exclusions cannot be reconstructed, and the revision does not supply a PRISMA flow diagram or an exhaustive inclusion count.

**Table 3:** Representative preclinical therapeutic evidence and preparation heterogeneity.

Study	Preparation and model	Biological evidence	Reported outcome	Interpretation limit
Hu et al. (17)	Embryonic stem cell (ESC)-sEVs; VaD rat. Differential ultracentrifugation; TEM, nano-flow particle analysis and EV/cellular marker panels.	Lysosome activation and NSC senescence	Cognition and neurogenesis improved	Selected cellular contamination controls do not establish all non-EV material absent; potency and chronic safety remain incomplete.
Ma et al. (18)	MSC-EVs; carotid-occlusion mouse. Size/morphology and CD63/CD29 characterization; particle-based dosing.	miR-132-3p perturbation and synaptic function	Memory and synaptic endpoints improved	Particle count does not prove purity; lipid-label uptake is not sufficient evidence of intact-vesicle cargo delivery.
Guy et al. (40)	MSC-EVs; hypertensive rat CSVD model. Serial centrifugation; NTA, TEM and EV/MSC surface-marker profiling.	Intranasal treatment; white matter protection	White matter and behavioral outcomes	Characterization supports an EV-enriched product, not equivalent potency or purity across MSC preparations.
Wang et al. (10)	hUCMSC-EVs; carotid-ligation VaD rat. Kit/ultracentrifugation workflow; TEM/NTA (46–150 nm), CD63/HSPA8/TSG101 and calnexin control.	LY294002-sensitive PI3K/AKT/Nrf2-associated signaling and microglial-marker changes	Behavioral and tissue protection	Unequal mortality; survivor selection and high-speed separation parameters unclear. Cargo-specific and Nrf2-specific causality, purity, batch potency and EV-specific attribution remain unresolved.
Yang et al. (9)	Microglial EVs after sinomenine treatment; hypoperfusion rat. Commercial kit; TEM, NTA, CD63/CD81.	miR-223-3p and inflammatory signaling	Inflammation and cognition improved	Kit-enriched material can retain non-EV components; drug carry-over and EV contribution require separation.
Wang et al. (7)	NSC-EVs; carotid-occlusion VaD mouse. Differential ultracentrifugation; NTA/TEM and CD63/TSG101; intranasal particle-based dosing.	miR-132-3p gain/loss; RASA1 reporter and overexpression in cultured neurons	miR-132-3p-enriched preparations improved cognitive and synaptic endpoints	Nestin capture and lipid-dye tracing do not prove lineage or intact-vesicle delivery. Corrigendum (62) replaced Golgi/DHE images and a beta-actin band.
Pan et al. (8)	Hypoxic NSC-EVs; VaD model. Differential ultracentrifugation; NTA, TEM, CD63/TSG101.	HIF-1α/RAB27A, miR-210 and SPRED1	Neuronal plasticity improved	Correction notice linked in the search record; independent replication and source-specific controls remain important.
Shen et al. (38)	Cyclic NGR peptide (cNGR)-engineered MSC-EVs; bilateral carotid stenosis (BCAS) mouse. Ultracentrifugation, particle/marker characterization; sucrose cushion for free-dye removal.	CD13-associated pericyte targeting	Cognition and vascular demyelination improved	Selected negative markers support characterization; manufacturing, off-target exposure and long-term safety remain unresolved.
Zheng et al. (60)	*L. salivarius* EV-metabolite platform; VaD model. Bacterial-derived preparation.	Lung-brain pathway	Barrier and inflammatory outcomes	Distinct product class; mammalian EV markers and safety assumptions cannot be transferred.
Qi et al. (41)	Neural stem cell EVs; VaD rat. Ultracentrifugation and morphology/particle assessment.	MIAT/miR-34b-5p/CALB1 perturbation	Learning and memory improved	Preclinical pathway evidence; long-term exposure, product purity and independent replication unresolved.
Yang et al. (34)	CCR2-enriched hUCMSC-EVs; post-stroke rat. Differential ultracentrifugation, TEM, qNano, CD9/CD63/CD81.	CCL2 binding and inflammatory-cell recruitment	Cognition, remyelination and oligodendrogenesis improved	Post-stroke model differs from chronic CSVD; shared manufacturing and functional-release standards absent.
Liu et al. (42)	Human induced pluripotent stem cell (iPSC)-derived MSC-sEVs; OGD/MCAO. Differential ultracentrifugation; TEM/NanoFCM (60–120 nm), CD9/CD63/TSG101 and GM130 control.	Reduced NSC senescence-associated phenotypes and increased neurogenic readouts	90-day working-memory and synaptic improvement after repeat dosing from 4 h post-MCAO	Active cargo and NSC-mediated cognitive causality unresolved; early treatment does not establish reversal of advanced VaD. Purity and product safety require further study.
Lin et al. (43)	Muscle/serum sEVs; hypoperfusion rat. Differential ultracentrifugation, TEM/NTA/WB and muscle-specific reporter tracing.	miR-17/20a-5p depletion, DEPTOR/mTOR perturbation	Exercise-associated synaptic and cognitive protection	Reporter and perturbation support source-pathway linkage; experimental efficacy is not a standardized therapeutic product.

Reported technical features are descriptive, not a retrospective MISEV compliance score. TEM, transmission electron microscopy; NTA, nanoparticle tracking analysis; WB, western blotting; NanoFCM, nanoflow cytometry; OGD, oxygen–glucose deprivation; MCAO, middle cerebral artery occlusion. An unlisted feature is not evidence that the source study omitted it. Supplementary material S2 identifies extraction limits.

**Table 4:** Phenotype-based design matrix for VCID EV studies.

Phenotype	NVU module	Preferred EV strategy	Candidate readout	Clinical endpoint	Principal risk
Post-stroke cognitive impairment	Inflammation, endothelium, recovery plasticity	Early biomarker study; staged recovery-product development	EV phenotype, NfL/GFAP, lesion evolution	Cognitive trajectory, function, recurrent events	Haemorrhagic transformation, thrombosis, immune activation
CSVD-dominant VCID	Endothelium, BBB, perivascular spaces, white matter	Imaging-linked EV profiling and longitudinal validation	EV proteins or miRNAs, endothelial EVs, MRI	WMH progression, lacunes, microbleeds, executive function	Assay variation, misclassification, vascular leakage
Chronic hypoperfusion or white matter injury	Pericytes, oligodendrocytes, mitochondria	Pericyte-targeted or myelin-protective products	Perfusion, myelin and source-specific cargo	Diffusion MRI, processing speed, executive function	Abnormal angiogenesis and off-target remodeling
Mixed AD and vascular pathology	Inflammation, oxidative stress, amyloid and tau overlap	Adjunctive stratification with AD biomarkers	EV omics plus amyloid, tau and vascular markers	Domain-specific cognition, imaging and function	Mechanistic confounding and attribution error
Advanced VaD	Multimodule NVU failure	Only after early-stage safety and exposure are established	Broad injury and function markers	ADL/IADL, caregiver burden, global cognition	Frailty, polypharmacy and limited reversibility

**Table 5:** Representative human EV biomarker studies and validation limitations.

Study	Cohort and design	Separation and assay	Finding and validation	Confounding or interpretation limit
Elahi et al. (36)	26 older adults; WMH 11, no WMH 15. Cross-sectional.	Plasma precipitation, CD31/CD146 enrichment; ELISA; CD81 normalization.	Age-adjusted AUC 0.82–0.89 for brain-enriched cargo; no independent validation.	Marker enrichment does not prove brain origin; small case–control sample.
van Kralingen et al. (48)	Discovery 39; qPCR validation 173. Stroke versus mimics, with SVD subtype analysis.	Serum precipitation; TEM/NTA; miRNA microarray and qPCR.	miR-17-family and miR-27b changes; separate validation cohort.	Acute-stroke diagnostic spectrum; not a chronic VCID cognitive-outcome test.
Zhao et al. (44)	40 CSVD-CI, 38 CSVD, 35 controls; single-center cross-sectional.	Commercial kit; TEM/NTA; RT-PCR, external miRNA control.	miR-223-3p association with CI, Hcy and CRP; no independent validation.	Comparative/correlation analyses; no multivariable cognitive-outcome model described. Text and table sample counts inconsistent.
Han et al. (19)	Discovery 7 VaD/7 controls; subsequent clinical verification and rat/cell work.	Serum ultracentrifugation; TEM/NTA; sequencing and RT-qPCR.	miR-154-5p associated with VaD; source paper reports clinical verification *n* = 23.	Small discovery cohort; clinical verification is not independent prospective validation; human origin/cargo causality unresolved.
Gao et al. (45)	150 CSVD, 80 controls; 16/16 used in pooled microarray discovery.	Plasma precipitation reagent; TEM, particle sizing and marker analysis; qPCR.	AUC 0.752; multivariable WMH association; no external validation.	Hypertension/smoking imbalance; model concerns imaging burden, not prospective cognitive prediction.
Qi et al. (47)	10 post-stroke cognitive impairment (PSCI) cases and 10 controls; exploratory cross-sectional.	Plasma TiO2 affinity enrichment; TEM/NTA, EV-marker WB; label-free proteomics.	259 differentially expressed proteins; no independent validation.	High-dimensional discovery in 20 participants; no validated decision threshold or adjusted predictive model.
Feng et al. (46)	18 CSVD-CI and 18 healthy controls; single exploratory case–control comparison.	PTM-EVS kit after centrifugation/filtration; TEM/NTA (112-nm peak); tandem mass tag (TMT) proteomics.	30 differentially expressed proteins (22 down, 8 up); no ROC or independent validation reported in the main article.	Heparin collection conflicts with serum wording. Replicate/pooling design and purity controls incompletely reported; no covariate-adjusted clinical model.
Yang et al. (50)	Discovery 100; validation reported as 211; longitudinal 68, mean 1.7 years.	Plasma affinity beads; TEM/particle analysis, TSG101/CD9/CD81 and calnexin; Liquid chromatography–tandem mass spectrometry (LC–MS/MS), then parallel reaction monitoring (PRM).	AUC 0.849–0.897 across distinct tasks; separate validation dataset and longitudinal risk analysis.	Subgroup/total counts inconsistent. Demographic/vascular covariates and internal calibration assessed; independent assay transfer/utility unproven.
You et al. (37)	Discovery 55; validation 291; longitudinal subset 87.	CSF CD31-positive small EV fraction; NTA and CD31/GLUT1 nano-flow cross-validation.	Validation VCI vs. AD AUC 0.762; with p-tau181 0.965. Separate cohort and longitudinal evidence.	Models include demographics, vascular risks and AD markers. Follow-up selection differs at baseline; source assignment and CSF scalability remain limitations.
Buntsma et al. (52)	155 emergency patients; 66 ischemic stroke, 89 other diagnoses.	Double-centrifuged plasma; calibrated immunophenotyping flow cytometry, 100–1,000 nm.	Clinical-plus-EV AUC 0.73 vs. 0.65; adjusted leukocyte-EV association.	Age/diastolic pressure modeled; acute setting, no chronic VCID validation; detected range exceeds sEV definition.

Discovery, verification, separate validation and prospective utility are distinct. AUCs concern different classification tasks and cannot rank studies directly. Source-reported inconsistencies are retained as limitations rather than corrected by inference.

### 2.3 Terminology and evidence appraisal

We use EV when vesicular material is reported but a size-defined fraction is not established. We use sEV or sEV-enriched preparation only as an operational size description supported by the source report. Exosome denotes demonstrated endosomal biogenesis; where this is unproven, the term appears only as the source authors’ terminology (25). Neither separation by precipitation or ultracentrifugation nor expression of CD9, CD63 or CD81 alone establishes endosomal origin or a homogeneous EV population. Older microparticle and microvesicle studies contribute context but are not pooled with sEV studies as an equivalent preparation class (26–30).

Methodological appraisal distinguishes the separation method, size distribution and particle measurement, orthogonal EV characterization, non-EV contaminants, cellular-attribution evidence and assay or functional controls (25). Morphology and particle counts alone do not identify every detected particle as an EV. EV-associated membrane and cytosolic markers support identity; calnexin or GM130 controls address selected cellular contaminants but do not exclude plasma lipoproteins or soluble proteins. ApoA1/ApoB, albumin and other matrix-appropriate controls, together with orthogonal separation, are relevant to purity. For functional claims, EV-depleted fractions, process-matched controls and cargo perturbation help distinguish vesicular effects from co-isolated material. Table 3 and Supplementary material S2 record reported technical features and interpretation limits; unavailable details are distinguished from evidence that a feature was not measured.

### 2.4 Use of AI-assisted tools

OpenAI Codex (OpenAI, San Francisco, CA, USA; GPT-5 series during original preparation in July–September 2026 and GPT-6 during revision in October 2026) supported literature retrieval, source extraction, language editing, revision correspondence and R-code preparation for the conceptual figure. These activities did not generate experimental data or validate the proposed evidence map. Verification of source details, scientific interpretation and approval of the final text remain the responsibility of the authors.

## 3 NVU modules connect EV mechanisms with clinical phenotypes

### 3.1 Inflammation and immune signaling

Inflammation-related EVs provide candidate measures of active disease biology. Chronic hypoperfusion, BBB injury and white matter damage activate microglia, border-associated macrophages, endothelial immune signaling and peripheral myeloid cells (31–33, 64). hUCMSC-EVs and miRNA-containing preparations modify microglial responses, NLRP3-related pyroptosis and PI3K/AKT/Nrf2 signaling in experimental models (9, 10). CCR2-enriched MSC-EVs also improve cognitive and remyelination outcomes after experimental stroke (34). Conversely, foam cell-derived EVs worsen ischemic white matter injury, and earlier hypoperfusion-associated microparticle studies report proapoptotic activity (26, 35). EV effects therefore depend on preparation, source and disease context.

Clinical studies should pair inflammatory cargo with a prespecified phenotype, such as persistent inflammation after stroke, CSVD-dominant white matter injury or chronic hypoperfusion. Candidate target-engagement panels may combine longitudinal EV cargo with NfL or GFAP, BBB permeability, white matter hyperintensity progression, executive function and processing speed. Safety assessment should include immune activation, excessive immunosuppression and procoagulant vesicle activity. The M1/M2 binary model does not adequately describe human microglial states and should be replaced by functional assays and cell-state analysis.

### 3.2 BBB, endothelium, pericytes and microvascular stability

Endothelial and pericyte biology links EV research to VCID (36–38, 65). Plasma endothelial-enriched preparations were associated with WMH and cognition in an early study of 26 functionally normal older adults (36). More recent CSF work includes distinct discovery and validation cohorts and longitudinal observations (37). These biomarker studies differ in matrix, enrichment and cellular-attribution evidence. Engineered pericyte-targeted EVs improve cognition and vascular demyelination in a chronic hypoperfusion mouse model (38). Microparticle findings are not uniformly positive. In 74 patients with recent lacunar infarcts and 74 matched controls, platelet-activation parameters did not differ, whereas endothelial markers did (39). Endogenous EV associations and therapeutic manipulation represent different claims and require different validation.

Vascular repair should be measured as functional microvascular stability rather than vessel number alone. Relevant outcomes include perfusion, BBB leakage, tight junction integrity, pericyte coverage, white matter structure and microhaemorrhage. Older patients with VCID frequently have hypertension, diabetes, atherosclerosis, antithrombotic exposure and cerebral microbleeds. Abnormal angiogenesis, coagulation and vascular leakage must therefore be assessed alongside efficacy.

### 3.3 Mitochondrial stress, oxidative injury and white matter integrity

Chronic hypoperfusion reduces the resilience of oligodendrocytes and axons through mitochondrial injury and oxidative stress. Stem cell-derived EVs modify Nrf2-related antioxidant responses, mitochondrial homeostasis, apoptosis and myelin injury in cell and animal models (10, 17, 18, 40, 63). Foam cell-derived exosomes also show that peripheral vesicles can injure the hypoperfused brain through source- and cargo-dependent mechanisms (35). Molecular and histological changes can support early target engagement, but improved maze performance does not establish human disease modification.

Clinical mapping should prioritize white matter hyperintensity volume and progression, diffusion measures, myelin-sensitive imaging, cerebral perfusion, executive function and processing speed. Mitochondrial or metabolic EV cargo should first be treated as a candidate biomarker and tested for analytical stability, source specificity and longitudinal predictive value in independent cohorts.

### 3.4 Neurogenesis, synaptic plasticity and network recovery

Neural, mesenchymal and embryonic stem cell-derived EV preparations modify neurite growth, dendritic spines, neurogenesis and cognition through miR-132-3p, miR-210 and lysosome-related pathways (7, 8, 17, 18). MIAT-containing neural stem cell EVs and induced MSC-sEVs provide additional preclinical recovery evidence (41, 42). Muscle-derived sEVs link exercise with hippocampal mammalian target of rapamycin (mTOR) signaling and cognitive protection in chronic hypoperfusion, supported by cargo depletion and pathway perturbation experiments (43). These findings broaden the source-cargo mechanisms under study, but do not establish interchangeable preparations or efficacy in patients.

This module also carries the greatest risk of over-extrapolation. Animal behavioral improvement does not show that advanced VaD is reversible. Translational studies require domain-specific cognitive testing, functional outcomes, prolonged follow-up and a target-engagement measure linked to network or synaptic biology. Disease stage should be defined before enrolment. Earlier disease may preserve greater plasticity, whereas advanced disease calls for endpoints centered on slowed decline, daily function and safety.

## 4 Patient stratification determines biomarkers, endpoints and risk

VCID heterogeneity makes VaD an inadequate undifferentiated enrolment label. Post-stroke cognitive impairment permits study of early inflammation, endothelial activation, recovery plasticity and cognitive trajectories. CSVD-dominant VCID is suited to biomarkers associated with white matter hyperintensities, lacunes, microbleeds and executive dysfunction. Chronic hypoperfusion and white matter injury provide a rationale for pericyte, endothelial, mitochondrial and myelin-directed strategies. Mixed AD and vascular pathology requires amyloid and tau context, while advanced VaD requires explicit consideration of frailty, polypharmacy and limited reversibility (1, 3–6).

Stratification variables should be defined before biospecimen collection or treatment allocation. The minimum set includes stroke timing and subtype, CSVD imaging burden, perfusion, BBB integrity, microbleeds, cognitive domains, AD co-pathology, inflammatory state and antithrombotic exposure. These variables determine EV sampling times, relevant cargo, imaging and cognitive outcomes, and safety monitoring (Table 4).

## 5 Human EV evidence and biomarker validation

Human EV studies differ in population, separation method, assay and validation design (Table 5). Early miRNA and proteomic reports are mainly exploratory, with small samples and limited external replication (19, 44–47). The 26-participant endothelial-enriched EV study used precipitation followed by CD31/CD146 immunoenrichment and age-adjusted analyses, but this approach does not itself prove brain-specific origin (36). A separate stroke study used a discovery cohort of 39 and a qPCR validation cohort of 173; its stroke-mimic comparison and SVD subtype findings are informative for assay design, not direct validation of chronic VCID diagnosis (48). Cerebral autosomal dominant arteriopathy with subcortical infarcts and leukoencephalopathy (CADASIL) EV work extends the vascular context to hereditary disease, but its 30-case/30-control design does not establish generalizability to sporadic VCID (49).

Yang et al. report a 100-participant proteomic discovery set, a separate targeted-proteomic validation dataset reported as 211, and a longitudinal CSVD subset of 68 followed for a mean of 1.7 years (50). The article contains inconsistent validation totals and subgroup counts, which should be resolved before quantitative synthesis. Antibody-affinity bead enrichment, EV marker analysis and particle characterization support a defined assay workflow, but do not establish brain-cell origin. The reported AUCs of 0.849–0.897 concern different classification tasks. The investigators assessed clinical covariates and reported internal calibration; independent laboratory transfer, a locked externally evaluated model and prospective decision impact remain to be established.

You et al. report a CSF discovery cohort of 55, a validation cohort of 291 and a longitudinal subset of 87 (37). Their brain endothelial-associated EV measure (c-BEEVs) is the proportion of CD31-positive small EVs among total CSF small EVs. CD31/GLUT1 nano-flow cytometry provides supporting phenotyping, but cellular attribution must be interpreted with the sample matrix and marker specificity. In the validation cohort, c-BEEVs alone discriminate vascular cognitive impairment (VCI) from AD with AUC 0.762 (95% CI 0.691–0.833); combining c-BEEVs with phosphorylated tau at threonine 181 (p-tau181) gives AUC 0.965 (95% CI 0.942–0.988). These task-specific results cannot be compared directly with plasma CSVD classification AUCs. Baseline cognition models adjust for demographic variables, vascular risks and AD biomarkers. Followed and non-followed participants differ on several baseline measures, so attrition and subgroup selection constrain longitudinal interpretation.

Clinical confounding and generalizability require explicit assessment. Age, sex, education, hypertension, diabetes, renal function, inflammation, medications, platelet activation, stroke timing and AD co-pathology can affect EV signals or cognitive outcomes. A longitudinal study of 197 patients with diabetes and acute stroke links astrocyte-enriched complement measures to later cognition, but its population and source-enrichment assumptions differ from sporadic CSVD (51). Buntsma et al. analyze 155 patients with stroke-like symptoms using calibrated flow cytometry over a reported 100–1,000 nm particle range (52). Their clinical-plus-EV AUC of 0.73 versus 0.65 supports acute-stroke assay development, not sEV-specific VCID diagnosis. Clinical utility requires a prospectively specified decision, a locked assay and evidence that use improves management or patient-relevant outcomes beyond established biomarkers and imaging (1, 4, 5).

Cellular attribution is a separate evidential claim from detection of an EV-associated signal. A circulating endothelial marker need not be brain-specific, and soluble marker fragments or protein aggregates can bind capture reagents. Demonstration of marker colocalization on intact particles, appropriate negative and isotype controls, separation from soluble fractions and orthogonal phenotyping strengthens attribution (25). CD31 is also expressed by platelets and leukocytes, and a CSF CD31-positive signal should be interpreted with matrix-specific controls and additional markers such as GLUT1 (37). Co-isolated lipoproteins, albumin, soluble cytokines or ribonucleoprotein complexes may contribute to proteomic or RNA signals. Serum clotting and platelet carry-over further complicate comparison with plasma. These uncertainties can coexist with a useful empirical assay association, but they constrain claims about biological origin and mechanism.

## 6 Therapeutic EVs require development as defined biological products

A therapeutic EV program must define its product and intended activity. The product description should specify source cells, culture and stimulation conditions, cargo, critical quality attributes, potency assay, purification, dose metric, route, biodistribution, stability and batch consistency (53–59). MSC, neural stem cell, endothelial, engineered pericyte-targeted and bacterial EV products carry different mechanisms and risks and should not be treated as one therapeutic class (7, 8, 18, 20, 38, 40, 60).

Potency assays should reproduce the proposed mechanism. A BBB-stabilizing product requires a relevant endothelial-pericyte assay with barrier and microvascular readouts. A myelin-protective product requires oligodendrocyte or white matter endpoints. A recovery product should connect synaptic or network measures with functional outcomes. *In vivo* studies should quantify brain exposure, peripheral accumulation, persistence, dose–response relationships and target engagement.

Natural origin does not establish safety. Hypertension, cerebral microbleeds, antithrombotic therapy, malignancy history, renal or hepatic dysfunction and polypharmacy can alter risk in older patients with VCID. Nonclinical and early clinical programs should assess immunogenicity, coagulation, abnormal angiogenesis, vascular leakage, microhaemorrhage, off-target uptake, tumor-promoting signals, organ toxicity and delayed effects. An early AD trial can inform monitoring but cannot replace validation in VCID (61).

## 7 Derivation and intended use of the five-domain evidence map

The map combines existing requirements rather than introducing a new theory of clinical readiness. Biological rationale draws on source-cargo-recipient and functional attribution principles; technical control draws on MISEV2023 and assay or product reproducibility requirements (25). Phenotype-relevant translation and clinical validation draw on biomarker-development and VCID imaging, cognition and trial guidance (1, 5, 6). Prospective evaluation draws on therapeutic-product identity, potency, exposure and safety requirements or, for biomarkers, a defined clinical-use question (55–59). Tables 3, 5 illustrate recurring evidence gaps. These sources motivate the domain content, but the five-domain grouping and its application to VCID are author choices, not empirically derived boundaries or validated thresholds (Table 6).

**Table 6:** Provenance and appraisal questions for five proposed evidence domains.

Domain	Provenance	Therapeutic appraisal	Biomarker appraisal
1 Biological rationale	MISEV attribution/function principles (25); mechanisms in Table 3	Defined preparation, reproducible biological activity and mechanism-linked potency; complete mechanism may remain unresolved.	Empirical relationship to intended phenotype; support cellular origin only to the extent directly tested.
2 Technical control	MISEV2023 (25); assay/product development (55–59)	Separation, identity, matrix-specific purity controls, dose metrics, functional controls and batch consistency.	Collection/processing, separation, repeatability, detection limits, contaminants and platform reproducibility.
3 Phenotype-relevant translation	VCID biomarker/trial guidance (1, 5, 6)	Appropriate model and disease stage, exposure, target engagement, functional outcomes and safety.	Defined population with imaging/cognitive linkage and longitudinal evidence where needed for intended use.
4 Clinical validation	Clinical biomarker and product development (1, 5, 6, 57, 59)	Human safety, exposure and mechanism-linked endpoints for the specified product and indication.	Separate validation, locked model/threshold, calibration and incremental value in the intended setting.
5 Prospective evaluation	Trial/utility preparation principles (1, 6, 59)	Executable early-phase protocol, prespecified eligibility/endpoints, risk monitoring and stopping rules.	Prospective study of a specified clinical decision, benefit, feasibility and patient-relevant outcomes.

Domain numbering is descriptive. There is no validated score, weighting, prerequisite hierarchy or automatic stop/advance rule. Requirements depend on intended use, and evidence may accumulate in parallel.

The domains organize questions for qualitative appraisal. They do not assign candidate scores, readiness levels or automatic advancement decisions. Evidence can accumulate in parallel, and the appropriate evidential requirement depends on intended use. A therapeutic candidate can have reproducible potency and *in vivo* efficacy before every source-cargo-recipient step is resolved; mechanistic uncertainty should guide further experiments and safety interpretation rather than automatically exclude that candidate. A biomarker may have empirical predictive validity without proving that its cargo causes disease, but a claim of cellular origin still requires direct support. Technical reproducibility and clinically relevant validation remain necessary to interpret either pathway.

VCID phenotype modifies interpretation in each domain. Studies should specify whether they address post-stroke impairment, sporadic CSVD, hereditary CSVD, hypoperfusion-related injury, mixed pathology or advanced dementia. Imaging, executive function, processing speed, daily function and safety monitoring must match that population. The map has not been developed through expert consensus, tested for inter-rater reproducibility or prospectively evaluated. Future evaluation should define the appraisal items and intended use, compare independent raters, assess feasibility and test whether the map improves evidence assessment or protocol design. Until then, it is a conceptual organizing tool for research discussion.

## 8 Discussion and research priorities

Three questions should receive priority. First, head-to-head comparisons of EV sources under shared isolation, characterisation, dose and potency conditions should determine whether source changes efficacy and risk. Second, longitudinal VCID cohorts should model EV signals jointly with MRI, cognitive domains, NfL, GFAP and AD co-pathology to test incremental value. Third, therapeutic programs should connect mechanism-matched potency assays with dose, biodistribution, target engagement and long-term safety.

Reporting should follow the entire evidence chain. Basic studies require MISEV-consistent information, randomization, blinding, prespecified primary outcomes and negative results. Biomarker studies require pre-analytical protocols, repeatability, cohort definitions, confounder control and external validation. Therapeutic studies require critical quality attributes, release criteria, potency, dose, distribution and toxicology. Negative dose–response findings, ineffective models and failed batches define the boundaries of a product and mechanism and should be reported.

Model and clinical labels must also be aligned. Bilateral carotid stenosis or occlusion models reproduce selected hypoperfusion, white matter and cognitive features but do not represent all VCID. Authors should identify the patient phenotype, time window and endpoint that each model approximates and should not translate improvement in rodent spatial learning into a claim of human disease modification.

### 8.1 Limitations of the evidence map and framework

This narrative review has several limitations. PubMed remains the principal database, so the expanded terminology and citation tracing reduce but do not eliminate retrieval bias. The original search and screening records were not retained, and the revision search cannot reconstruct them. Representative selection and AI-assisted extraction are vulnerable to judgment and transcription errors; no independent duplicate screening, formal risk-of-bias assessment or meta-analysis was performed. EV size distributions, separation methods, contaminants and cellular-attribution evidence differ across studies, preventing treatment of their preparations as one sEV class. Separate validation datasets or follow-up within a research program do not establish cross-laboratory reproducibility or prospective clinical utility. Finally, the author-proposed evidence map has no consensus, inter-rater or prospective validation and should not be used as a readiness score or a regulatory decision rule.

## 9 Conclusion

EV research links intercellular communication, candidate biomarkers and therapeutic delivery across VCID phenotypes. The strongest recurring biological themes involve inflammation, BBB and microvascular stability, mitochondrial and white matter injury, and neural plasticity. Therapeutic evidence remains predominantly preclinical. Some human studies provide separate validation cohorts and longitudinal associations, but assay comparability, cellular attribution and prospective clinical utility remain unresolved.

The neurovascular evidence map relates existing EV and clinical-development requirements to defined patient populations, imaging, cognition, assay performance, product attributes and safety. Its contribution is integrative, and its five domains remain unvalidated. Separate biomarker and therapeutic pathways help identify the evidence needed for a specific claim without interpreting a clinical association as a treatment mechanism or an animal behavioral effect as human efficacy.

## Glossary

AD: Alzheimer disease
ADL: activities of daily living
AUC: area under the receiver operating characteristic curve
BBB: blood–brain barrier
CRP: C-reactive protein
CSF: cerebrospinal fluid
CSVD: cerebral small vessel disease
EPC: endothelial progenitor cell
EV: extracellular vesicle
GFAP: glial fibrillary acidic protein
Hcy: homocysteine
hUCMSC: human umbilical cord mesenchymal stromal cell
IADL: instrumental activities of daily living
MSC: mesenchymal stromal cell
NfL: neurofilament light chain
NLRP3: NOD-like receptor protein 3
NSC: neural stem cell
NVU: neurovascular unit
sEV: small extracellular vesicle
VaD: vascular dementia
VCID: vascular cognitive impairment and dementia
WMH: white matter hyperintensity
