# Supplementary material for: Extracellular vesicles in vascular cognitive impairment and dementia: a neurovascular evidence map for patient stratification and clinical translation
Source: Front Neurol. 2026 Oct 30;17:1986081. doi: 10.3389/fneur.2026.1986081 (PMC13760277; doi:10.3389/fneur.2026.1986081)
Supplement: Supplementary file 1 [file Data_Sheet_1.PDF]

Supplementary Material S1 revision search record

Revision-stage PubMed retrieval record

Search executed 8 October 2026. Publication-date range 1 January 1900 to 8 October 2026. Database PubMed. Return count 140.

This is a new revision search. The original July 2026 export and screening log are unavailable. The disposition notes identify use in this representative narrative map or relevance to its scope; they are not a systematic full-text eligibility ledger. Title/abstract triage and methodological extraction were AI-assisted. Independent duplicate screening was not performed.

("Extracellular Vesicles"[MeSH] OR "extracellular vesicle*"[tiab] OR exosome*[tiab] OR microvesicle*[tiab] OR microparticle*[tiab] OR ectosome*[tiab] OR "small EV*"[tiab] OR "extracellular particle*"[tiab]) AND ("Dementia, Vascular"[MeSH] OR "vascular cognitive impairment"[tiab] OR "vascular cognitive impairment and dementia"[tiab] OR "vascular dementia"[tiab] OR "cerebral small vessel disease"[tiab] OR "small vessel disease"[tiab] OR "chronic cerebral hypoperfusion"[tiab] OR "cerebral hypoperfusion"[tiab] OR "post-stroke cognitive impairment"[tiab] OR "poststroke cognitive impairment"[tiab] OR ("white matter"[tiab] AND (ischemi*[tiab] OR ischaemi*[tiab] OR hypoperfus*[tiab]))) AND ("1900/01/01"[Date - Publication] : "2026/10/08"[Date - Publication])

1. PMID 42781292

Bidirectional brain-heart communication in coronary heart disease: from coronary plaques to central autonomic networks.

Disposition: Outside the primary narrative evidence scope. Broad background or indirect relevance; outside the representative primary evidence set.

Source: https://pubmed.ncbi.nlm.nih.gov/42781292/

2. PMID 42777603

Post-stroke neuroplasticity: Spatiotemporal multicellular networks and traditional Chinese medicine interventions.

Disposition: Outside the primary narrative evidence scope. Broad background or indirect relevance; outside the representative primary evidence set.

Source: https://pubmed.ncbi.nlm.nih.gov/42777603/

3. PMID 42776738

Exosomal lncRNAs in Cerebrovascular Diseases: Biomarkers, Pathological Mechanisms, and Therapeutic Potential.

Disposition: Contextual or additional relevant record. Relevant to mechanisms, vascular phenotype, methods or related literature; not all such records are cited or tabled in this narrative selection.

Source: https://pubmed.ncbi.nlm.nih.gov/42776738/

4. PMID 42666340

Biomarkers of perioperative neurocognitive disorders after cardiac surgery: from mechanistic insights to clinical translation.

Disposition: Outside the primary narrative evidence scope. Broad background or indirect relevance; outside the representative primary evidence set.

Source: https://pubmed.ncbi.nlm.nih.gov/42666340/

5. PMID 42664679

Biomarkers of the blood-brain barrier and the neurovascular unit and their relationship with cognitive decline and cognitive frailty.

Disposition: Contextual or additional relevant record. Relevant to mechanisms, vascular phenotype, methods or related literature; not all such records are cited or tabled in this narrative selection.

Source: https://pubmed.ncbi.nlm.nih.gov/42664679/

6. PMID 42645689

Exosomes: Mechanisms, Diagnosis, and Therapeutic Progress in Vascular Cognitive Impairment.

Disposition: Contextual or additional relevant record. Relevant to mechanisms, vascular phenotype, methods or related literature; not all such records are cited or tabled in this narrative selection.

Source: https://pubmed.ncbi.nlm.nih.gov/42645689/

7. PMID 42554250

Early identification of vascular cognitive impairment from a multimodal perspective: a combined diagnosis from targeted cognitive assessments, imaging biomarkers, and molecular fluid biomarkers to ecological behavioral characteristics.

Disposition: Contextual or additional relevant record. Relevant to mechanisms, vascular phenotype, methods or related literature; not all such records are cited or tabled in this narrative selection.

Source: https://pubmed.ncbi.nlm.nih.gov/42554250/

8. PMID 42471674

Correction: NSC-derived extracellular vesicles-mediates neuronal plasticity enhancement in vascular dementia via transferring miR-210.

Disposition: Correction notice linked to source study. Check the corrected source version; do not count as a separate efficacy study.

Source: https://pubmed.ncbi.nlm.nih.gov/42471674/

9. PMID 42365442

Fractal Complexity of the Circle of Willis Links Circulating Microparticles to Silent Cerebral Small Vessel Disease.

Disposition: Contextual or additional relevant record. Relevant to mechanisms, vascular phenotype, methods or related literature; not all such records are cited or tabled in this narrative selection.

Source: https://pubmed.ncbi.nlm.nih.gov/42365442/

10. PMID 42278362

Stem Cell-Derived Extracellular Vesicles Ameliorate the Neuron Mitochondrial Damage Induced by ROS-, LPS-Exposure: In Vitro Model of Neuron, Microglia, and Astrocyte Triple Co-Culture.

Disposition: Contextual or additional relevant record. Relevant to mechanisms, vascular phenotype, methods or related literature; not all such records are cited or tabled in this narrative selection.

Source: https://pubmed.ncbi.nlm.nih.gov/42278362/

11. PMID 42226579

Engineered Pericyte-Targeted Extracellular Vesicles Protect Against Hypoperfusion-Induced Cognitive Impairment and Vascular Demyelination.

Disposition: Included in a representative primary evidence table. Direct disease/model relevance or explicitly identified acute-stroke assay context.

Source: https://pubmed.ncbi.nlm.nih.gov/42226579/

12. PMID 42184678

Non-coding RNA regulatory networks in post-stroke neuropsychiatric complications: Mechanisms, delivery strategies, and translational challenges.

Disposition: Contextual or additional relevant record. Relevant to mechanisms, vascular phenotype, methods or related literature; not all such records are cited or tabled in this narrative selection.

Source: https://pubmed.ncbi.nlm.nih.gov/42184678/

13. PMID 42179845

Mesenchymal stromal/stem cell-derived extracellular vesicles in brain disorders: mechanisms of repair and recovery.

Disposition: Contextual or additional relevant record. Relevant to mechanisms, vascular phenotype, methods or related literature; not all such records are cited or tabled in this narrative selection.

Source: https://pubmed.ncbi.nlm.nih.gov/42179845/

14. PMID 42150695

Peripheral biomarkers of neuroinflammation in Alzheimer's disease and vascular dementia.

Disposition: Contextual or additional relevant record. Relevant to mechanisms, vascular phenotype, methods or related literature; not all such records are cited or tabled in this narrative selection.

Source: https://pubmed.ncbi.nlm.nih.gov/42150695/

15. PMID 42142143

Neural stem cell extracellular vesicle-mediated delivery of astragaloside IV attenuates hypoxic-ischemic brain damage by activating the mTOR pathway.

Disposition: Outside the primary narrative evidence scope. Neonatal/perinatal or pediatric injury differs from adult VCID.

Source: https://pubmed.ncbi.nlm.nih.gov/42142143/

16. PMID 42129839

Plasma extracellular vesicle proteins biomarker for cerebral small vessel disease related cognitive impairment.

Disposition: Included in a representative primary evidence table. Direct disease/model relevance or explicitly identified acute-stroke assay context.

Source: https://pubmed.ncbi.nlm.nih.gov/42129839/

17. PMID 42121153

The lung-brain axis mediates the neuroprotective effects of nasally administered L. salivarius and its EV-delivered metabolite in vascular dementia.

Disposition: Included in a representative primary evidence table. Direct disease/model relevance or explicitly identified acute-stroke assay context.

Source: https://pubmed.ncbi.nlm.nih.gov/42121153/

18. PMID 42120733

Brain endothelial cell-derived extracellular vesicles (c-BEEVs) as a promising biomarker for brain vascular pathology and cognitive decline.

Disposition: Included in a representative primary evidence table. Direct disease/model relevance or explicitly identified acute-stroke assay context.

Source: https://pubmed.ncbi.nlm.nih.gov/42120733/

19. PMID 42099154

Demyelination in Vascular Dementia: Focus on Oligodendrocytes, Microglia, and Their Interaction.

Disposition: Contextual or additional relevant record. Relevant to mechanisms, vascular phenotype, methods or related literature; not all such records are cited or tabled in this narrative selection.

Source: https://pubmed.ncbi.nlm.nih.gov/42099154/

20. PMID 41990907

Altered perivascular diffusion and elevated circulating cell-derived microparticles among individuals with enlarged perivascular spaces in subclinical cerebral small vessel disease.

Disposition: Contextual or additional relevant record. Relevant to mechanisms, vascular phenotype, methods or related literature; not all such records are cited or tabled in this narrative selection.

Source: https://pubmed.ncbi.nlm.nih.gov/41990907/

21. PMID 41979529

Case of Broca's Aphasia as an expression of cerebral decompression sickness with advanced non-invasive monitoring during HBO_2 treatment.

Disposition: Outside the primary narrative evidence scope. Different condition or outcome without direct VCID/white-matter EV evidence for the primary map.

Source: https://pubmed.ncbi.nlm.nih.gov/41979529/

22. PMID 41897300

Neurovascular Unit-Derived Extracellular Vesicles as Regulators of Post-Stroke Pathology and Neurorestoration.

Disposition: Contextual or additional relevant record. Relevant to mechanisms, vascular phenotype, methods or related literature; not all such records are cited or tabled in this narrative selection.

Source: https://pubmed.ncbi.nlm.nih.gov/41897300/

23. PMID 41819672

Ginkgolide B attenuates post-stroke cognitive impairment via exosomal miR-299a-3p regulation of the TRIL/PI3K/AKT pathway.

Disposition: Outside the primary narrative evidence scope. Broad background or indirect relevance; outside the representative primary evidence set.

Source: https://pubmed.ncbi.nlm.nih.gov/41819672/

24. PMID 41788687

The multi-target protective effects of quercetin in cerebrovascular diseases: a dietary strategy for endothelial repair and neuroprotection.

Disposition: Outside the primary narrative evidence scope. Broad background or indirect relevance; outside the representative primary evidence set.

Source: https://pubmed.ncbi.nlm.nih.gov/41788687/

25. PMID 41750303

Neurovascular Impairment in Type 2 Diabetes Mellitus: The Role of Adipocyte-Derived Exosomes.

Disposition: Contextual or additional relevant record. Relevant to mechanisms, vascular phenotype, methods or related literature; not all such records are cited or tabled in this narrative selection.

Source: https://pubmed.ncbi.nlm.nih.gov/41750303/

26. PMID 41722741

Exosome-mediated neuroprotection in vascular dementia: Mechanisms, molecular pathways, and therapeutic prospects.

Disposition: Contextual or additional relevant record. Relevant to mechanisms, vascular phenotype, methods or related literature; not all such records are cited or tabled in this narrative selection.

Source: https://pubmed.ncbi.nlm.nih.gov/41722741/

27. PMID 41642667

Time-dependent neurovascular unit dysfunction in ischemic stroke: mechanisms of neurovascular uncoupling and its clinical impact.

Disposition: Contextual or additional relevant record. Relevant to mechanisms, vascular phenotype, methods or related literature; not all such records are cited or tabled in this narrative selection.

Source: https://pubmed.ncbi.nlm.nih.gov/41642667/

28. PMID 41622493

Immune cells in cerebral small vessel disease: Emerging roles and potential targeting therapies.

Disposition: Contextual or additional relevant record. Relevant to mechanisms, vascular phenotype, methods or related literature; not all such records are cited or tabled in this narrative selection.

Source: https://pubmed.ncbi.nlm.nih.gov/41622493/

29. PMID 41595697

From Cerebrovascular Injury to Vascular Cognitive Impairment and Dementia: Therapeutic Potential of Stem Cell-Derived Extracellular Vesicles.

Disposition: Contextual or additional relevant record. Relevant to mechanisms, vascular phenotype, methods or related literature; not all such records are cited or tabled in this narrative selection.

Source: https://pubmed.ncbi.nlm.nih.gov/41595697/

30. PMID 41595595

Oxidative Stress as a Central Mechanistic Bridge Between Alzheimer's and Vascular Pathologies in Mixed Dementia: Emerging Evidence and Therapeutic Perspectives.

Disposition: Contextual or additional relevant record. Relevant to mechanisms, vascular phenotype, methods or related literature; not all such records are cited or tabled in this narrative selection.

Source: https://pubmed.ncbi.nlm.nih.gov/41595595/

31. PMID 41559842

Brain and circulating EV proteome signatures in schizophrenia as prognostic markers for age-related dementia.

Disposition: Outside the primary narrative evidence scope. Different condition or outcome without direct VCID/white-matter EV evidence for the primary map.

Source: https://pubmed.ncbi.nlm.nih.gov/41559842/

32. PMID 41510820

Therapeutic effect of amniotic membrane mesenchymal stem cell-derived exosome-rich conditioned medium in cerebral palsy model.

Disposition: Outside the primary narrative evidence scope. Neonatal/perinatal or pediatric injury differs from adult VCID.

Source: https://pubmed.ncbi.nlm.nih.gov/41510820/

33. PMID 41304786

Nanoparticle-Mediated Nose-to-Brain Delivery for Ischemic Stroke Therapy: Preclinical Insights.

Disposition: Outside the primary narrative evidence scope. Broad background or indirect relevance; outside the representative primary evidence set.

Source: https://pubmed.ncbi.nlm.nih.gov/41304786/

34. PMID 41294823

WHITE MATTER MATTERS: New Approach to the Brain's Hidden Half Using Circulating Oligodendrocyte-Derived Extracellular Vesicles.

Disposition: Contextual or additional relevant record. Relevant to mechanisms, vascular phenotype, methods or related literature; not all such records are cited or tabled in this narrative selection.

Source: https://pubmed.ncbi.nlm.nih.gov/41294823/

35. PMID 41272428

DPSCs-Exos promote OPCs differentiation and white matter repair via Myl9-mediated PRMT5 nucleation after ischemic stroke.

Disposition: Contextual or additional relevant record. Relevant to mechanisms, vascular phenotype, methods or related literature; not all such records are cited or tabled in this narrative selection.

Source: https://pubmed.ncbi.nlm.nih.gov/41272428/

36. PMID 41237903

Oral Microbiome-Derived Proteins in Brain Extracellular Vesicles Circulate and Tie to Specific Dysbiotic and Neuropathological Profiles in Age-Related Dementias.

Disposition: Contextual or additional relevant record. Relevant to mechanisms, vascular phenotype, methods or related literature; not all such records are cited or tabled in this narrative selection.

Source: https://pubmed.ncbi.nlm.nih.gov/41237903/

37. PMID 41175992

White matter injury in young rats with cerebral palsy: the role of massage in regulating exosomes via the chemokine axis.

Disposition: Outside the primary narrative evidence scope. Neonatal/perinatal or pediatric injury differs from adult VCID.

Source: https://pubmed.ncbi.nlm.nih.gov/41175992/

38. PMID 41059755

Retraction Note.

Disposition: Outside the primary narrative evidence scope. Retraction notice, not a primary eligible EV result.

Source: https://pubmed.ncbi.nlm.nih.gov/41059755/

39. PMID 40962524

[Exosomes derived from mesenchymal stem cells alleviate white matter damage in neonatal rats by targeting the NLRP3 inflammasome].

Disposition: Outside the primary narrative evidence scope. Neonatal/perinatal or pediatric injury differs from adult VCID.

Source: https://pubmed.ncbi.nlm.nih.gov/40962524/

40. PMID 40901978

Crossing Pathological Boundaries: Multi-Target Restoration of the Neurovascular Unit in Alzheimer's and Vascular Dementia-From Modern Therapeutics to Traditional Chinese Medicine.

Disposition: Contextual or additional relevant record. Relevant to mechanisms, vascular phenotype, methods or related literature; not all such records are cited or tabled in this narrative selection.

Source: https://pubmed.ncbi.nlm.nih.gov/40901978/

41. PMID 40887468

Arterial stiffness and vascular aging: mechanisms, prevention, and therapy.

Disposition: Outside the primary narrative evidence scope. Different condition or outcome without direct VCID/white-matter EV evidence for the primary map.

Source: https://pubmed.ncbi.nlm.nih.gov/40887468/

42. PMID 40769126

Foam cell-derived exosomes: Messengers between atherosclerosis and microglia.

Disposition: Contextual or additional relevant record. Relevant to mechanisms, vascular phenotype, methods or related literature; not all such records are cited or tabled in this narrative selection.

Source: https://pubmed.ncbi.nlm.nih.gov/40769126/

43. PMID 40718100

Emerging biomarkers and frontier therapies: unveiling the role of endothelial dysfunction in cerebral small vessel disease.

Disposition: Contextual or additional relevant record. Relevant to mechanisms, vascular phenotype, methods or related literature; not all such records are cited or tabled in this narrative selection.

Source: https://pubmed.ncbi.nlm.nih.gov/40718100/

44. PMID 40635095

NSC-derived extracellular vesicles-mediates neuronal plasticity enhancement in vascular dementia via transferring miR-210.

Disposition: Included in a representative primary evidence table. Direct disease/model relevance or explicitly identified acute-stroke assay context.

Source: https://pubmed.ncbi.nlm.nih.gov/40635095/

45. PMID 40542975

The future of biomarkers for vascular contributions to cognitive impairment and dementia (VCID): proceedings of the 2025 annual workshop of the Albert research institute for white matter and cognition.

Disposition: Contextual or additional relevant record. Relevant to mechanisms, vascular phenotype, methods or related literature; not all such records are cited or tabled in this narrative selection.

Source: https://pubmed.ncbi.nlm.nih.gov/40542975/

46. PMID 40521199

An AIM2 inflammasome biomimetic mineralization inhibitor for vascular dementia therapy.

Disposition: Contextual or additional relevant record. Relevant to mechanisms, vascular phenotype, methods or related literature; not all such records are cited or tabled in this narrative selection.

Source: https://pubmed.ncbi.nlm.nih.gov/40521199/

47. PMID 40371544

Blood-Brain Barrier (BBB) Dysfunction in CNS Diseases: Paying Attention to Pericytes.

Disposition: Contextual or additional relevant record. Relevant to mechanisms, vascular phenotype, methods or related literature; not all such records are cited or tabled in this narrative selection.

Source: https://pubmed.ncbi.nlm.nih.gov/40371544/

48. PMID 40345179

The foam cell-derived exosomes exacerbate ischemic white matter injury via transmitting metabolic defects to microglia.

Disposition: Contextual or additional relevant record. Relevant to mechanisms, vascular phenotype, methods or related literature; not all such records are cited or tabled in this narrative selection.

Source: https://pubmed.ncbi.nlm.nih.gov/40345179/

49. PMID 40271743

Muscle-Derived Small Extracellular Vesicles Mediate Exercise-Induced Cognitive Protection in Chronic Cerebral Hypoperfusion.

Disposition: Included in a representative primary evidence table. Direct disease/model relevance or explicitly identified acute-stroke assay context.

Source: https://pubmed.ncbi.nlm.nih.gov/40271743/

50. PMID 40136659

Therapeutic Approaches and Potential Mechanisms of Small Extracellular Vesicles in Treating Vascular Dementia.

Disposition: Contextual or additional relevant record. Relevant to mechanisms, vascular phenotype, methods or related literature; not all such records are cited or tabled in this narrative selection.

Source: https://pubmed.ncbi.nlm.nih.gov/40136659/

51. PMID 40113668

Cerebromicrovascular senescence in vascular cognitive impairment: does accelerated microvascular aging accompany atherosclerosis?

Disposition: Contextual or additional relevant record. Relevant to mechanisms, vascular phenotype, methods or related literature; not all such records are cited or tabled in this narrative selection.

Source: https://pubmed.ncbi.nlm.nih.gov/40113668/

52. PMID 40113008

Neural stem cell-derived exosomes improve neurite outgrowth and cognitive function through transferring miR-132-3p.

Disposition: Included in a representative primary evidence table. Direct disease/model relevance or explicitly identified acute-stroke assay context.

Source: https://pubmed.ncbi.nlm.nih.gov/40113008/

53. PMID 40045356

Sinomenine alleviates neuroinflammation in chronic cerebral hypoperfusion by promoting M2 microglial polarization and inhibiting neuronal pyroptosis via exosomal miRNA-223-3p.

Disposition: Included in a representative primary evidence table. Direct disease/model relevance or explicitly identified acute-stroke assay context.

Source: https://pubmed.ncbi.nlm.nih.gov/40045356/

54. PMID 39859943

The Microbiota-Gut-Brain Axis: Key Mechanisms Driving Glymphopathy and Cerebral Small Vessel Disease.

Disposition: Outside the primary narrative evidence scope. Broad background or indirect relevance; outside the representative primary evidence set.

Source: https://pubmed.ncbi.nlm.nih.gov/39859943/

55. PMID 39751866

Aerobic Exercise Improves Cognitive Recovery in Mice with Chronic Cerebral Hypoperfusion by Modulating the Annexin-A1-MAPK Axis and Astrocyte Polarization.

Disposition: Contextual or additional relevant record. Relevant to mechanisms, vascular phenotype, methods or related literature; not all such records are cited or tabled in this narrative selection.

Source: https://pubmed.ncbi.nlm.nih.gov/39751866/

56. PMID 39719419

Pericytes and Extracellular Vesicle Interactions in Neurovascular Adaptation to Chronic Arterial Hypertension.

Disposition: Contextual or additional relevant record. Relevant to mechanisms, vascular phenotype, methods or related literature; not all such records are cited or tabled in this narrative selection.

Source: https://pubmed.ncbi.nlm.nih.gov/39719419/

57. PMID 39468528

Magnetic targeting enhances the neuroprotective function of human mesenchymal stem cell-derived iron oxide exosomes by delivering miR-1228-5p.

Disposition: Contextual or additional relevant record. Relevant to mechanisms, vascular phenotype, methods or related literature; not all such records are cited or tabled in this narrative selection.

Source: https://pubmed.ncbi.nlm.nih.gov/39468528/

58. PMID 39418518

Proteomic Spectrum of Serum Exosomes in Ischemic Stroke Patients Is Associated with Cognitive Impairment in the Post-Stroke Period.

Disposition: Contextual or additional relevant record. Relevant to mechanisms, vascular phenotype, methods or related literature; not all such records are cited or tabled in this narrative selection.

Source: https://pubmed.ncbi.nlm.nih.gov/39418518/

59. PMID 39148704

Reviewing migraine-associated pathophysiology and its impact on elevated stroke risk.

Disposition: Outside the primary narrative evidence scope. Different condition or outcome without direct VCID/white-matter EV evidence for the primary map.

Source: https://pubmed.ncbi.nlm.nih.gov/39148704/

60. PMID 38980986

Regulation of metabolism and inflammation: Links with oral and systemic health: Part 3-Emerging concepts from pathogenesis to therapy.

Disposition: Outside the primary narrative evidence scope. Different condition or outcome without direct VCID/white-matter EV evidence for the primary map.

Source: https://pubmed.ncbi.nlm.nih.gov/38980986/

61. PMID 38845728

Mechanism of Post-stroke Axonal Outgrowth and Functional Recovery.

Disposition: Contextual or additional relevant record. Relevant to mechanisms, vascular phenotype, methods or related literature; not all such records are cited or tabled in this narrative selection.

Source: https://pubmed.ncbi.nlm.nih.gov/38845728/

62. PMID 38669534

Complement Proteins in Serum Astrocyte-Derived Exosomes Are Associated with Poststroke Cognitive Impairment in Type 2 Diabetes Mellitus Patients.

Disposition: Contextual or additional relevant record. Relevant to mechanisms, vascular phenotype, methods or related literature; not all such records are cited or tabled in this narrative selection.

Source: https://pubmed.ncbi.nlm.nih.gov/38669534/

63. PMID 38645567

Mitigating the impact of mechanisms causing neuronal degeneration.

Disposition: Outside the primary narrative evidence scope. Different condition or outcome without direct VCID/white-matter EV evidence for the primary map.

Source: https://pubmed.ncbi.nlm.nih.gov/38645567/

64. PMID 38570800

Exploring vascular contributions to cognitive impairment and dementia (ENIGMA): protocol for a prospective observational study.

Disposition: Contextual or additional relevant record. Relevant to mechanisms, vascular phenotype, methods or related literature; not all such records are cited or tabled in this narrative selection.

Source: https://pubmed.ncbi.nlm.nih.gov/38570800/

65. PMID 38534387

All but Small: miRNAs from Wharton's Jelly-Mesenchymal Stromal Cell Small Extracellular Vesicles Rescue Premature White Matter Injury after Intranasal Administration.

Disposition: Outside the primary narrative evidence scope. Neonatal/perinatal or pediatric injury differs from adult VCID.

Source: https://pubmed.ncbi.nlm.nih.gov/38534387/

66. PMID 38513760

Neonatal Hypoxia-Ischemia alters Brain-Derived Contactin-2-Positive Extracellular Vesicles in the Mouse Plasma.

Disposition: Outside the primary narrative evidence scope. Neonatal/perinatal or pediatric injury differs from adult VCID.

Source: https://pubmed.ncbi.nlm.nih.gov/38513760/

67. PMID 38478127

Induced Mesenchymal Stem Cells-Small Extracellular Vesicles Alleviate Post-stroke Cognitive Impairment by Rejuvenating Senescence of Neural Stem Cells.

Disposition: Included in a representative primary evidence table. Direct disease/model relevance or explicitly identified acute-stroke assay context.

Source: https://pubmed.ncbi.nlm.nih.gov/38478127/

68. PMID 38423190

The role of neuroglial cells communication in ischemic stroke.

Disposition: Contextual or additional relevant record. Relevant to mechanisms, vascular phenotype, methods or related literature; not all such records are cited or tabled in this narrative selection.

Source: https://pubmed.ncbi.nlm.nih.gov/38423190/

69. PMID 38389559

Emerging role of mesenchymal stem cells-derived extracellular vesicles in vascular dementia.

Disposition: Contextual or additional relevant record. Relevant to mechanisms, vascular phenotype, methods or related literature; not all such records are cited or tabled in this narrative selection.

Source: https://pubmed.ncbi.nlm.nih.gov/38389559/

70. PMID 38339164

Immunological Profile and Markers of Endothelial Dysfunction in Elderly Patients with Cognitive Impairments.

Disposition: Contextual or additional relevant record. Relevant to mechanisms, vascular phenotype, methods or related literature; not all such records are cited or tabled in this narrative selection.

Source: https://pubmed.ncbi.nlm.nih.gov/38339164/

71. PMID 38211879

Identification of differentially expressed exosome proteins in serum as potential biomarkers for cognitive impairments in cerebral small vessel disease.

Disposition: Included in a representative primary evidence table. Direct disease/model relevance or explicitly identified acute-stroke assay context.

Source: https://pubmed.ncbi.nlm.nih.gov/38211879/

72. PMID 38132326

Proteome Profiling of Brain Vessels in a Mouse Model of Cerebrovascular Pathology.

Disposition: Contextual or additional relevant record. Relevant to mechanisms, vascular phenotype, methods or related literature; not all such records are cited or tabled in this narrative selection.

Source: https://pubmed.ncbi.nlm.nih.gov/38132326/

73. PMID 37975212

Cerebral uptake of microvesicles occurs in normocapnic but not hypocapnic passive hyperthermia in young healthy male adults.

Disposition: Outside the primary narrative evidence scope. Different condition or outcome without direct VCID/white-matter EV evidence for the primary map.

Source: https://pubmed.ncbi.nlm.nih.gov/37975212/

74. PMID 37828646

Targeting accelerated pulmonary ageing to treat chronic obstructive pulmonary disease-induced neuropathological comorbidities.

Disposition: Outside the primary narrative evidence scope. Different condition or outcome without direct VCID/white-matter EV evidence for the primary map.

Source: https://pubmed.ncbi.nlm.nih.gov/37828646/

75. PMID 37771642

Exosomal miR-320e through wnt2targeted inhibition of the Wnt/β-catenin pathway allevisate cerebral small vessel disease and cognitive impairment.

Disposition: Contextual or additional relevant record. Relevant to mechanisms, vascular phenotype, methods or related literature; not all such records are cited or tabled in this narrative selection.

Source: https://pubmed.ncbi.nlm.nih.gov/37771642/

76. PMID 37439367

Serum microRNA-501-3p is a potential diagnostic tool for detecting mild cognitive impairment: Ehime genome study.

Disposition: Outside the primary narrative evidence scope. Non-EV analyte, drug-only outcome, or an assay reagent termed microparticle rather than EV biology.

Source: https://pubmed.ncbi.nlm.nih.gov/37439367/

77. PMID 37374328

Brain Endothelial Cells Play a Central Role in the Development of Enlarged Perivascular Spaces in the Metabolic Syndrome.

Disposition: Contextual or additional relevant record. Relevant to mechanisms, vascular phenotype, methods or related literature; not all such records are cited or tabled in this narrative selection.

Source: https://pubmed.ncbi.nlm.nih.gov/37374328/

78. PMID 37211381

Plasma exosome proteomics reveals the pathogenesis mechanism of post-stroke cognitive impairment.

Disposition: Included in a representative primary evidence table. Direct disease/model relevance or explicitly identified acute-stroke assay context.

Source: https://pubmed.ncbi.nlm.nih.gov/37211381/

79. PMID 36968490

Therapeutic effects of CD133 + Exosomes on liver function after stroke in type 2 diabetic mice.

Disposition: Outside the primary narrative evidence scope. Different condition or outcome without direct VCID/white-matter EV evidence for the primary map.

Source: https://pubmed.ncbi.nlm.nih.gov/36968490/

80. PMID 36941024

Neuroprotective mechanism of human umbilical cord mesenchymal stem cell-derived extracellular vesicles improving the phenotype polarization of microglia via the PI3K/AKT/Nrf2 pathway in vascular dementia.

Disposition: Included in a representative primary evidence table. Direct disease/model relevance or explicitly identified acute-stroke assay context.

Source: https://pubmed.ncbi.nlm.nih.gov/36941024/

81. PMID 36921779

The NG2-glia is a potential target to maintain the integrity of neurovascular unit after acute ischemic stroke.

Disposition: Contextual or additional relevant record. Relevant to mechanisms, vascular phenotype, methods or related literature; not all such records are cited or tabled in this narrative selection.

Source: https://pubmed.ncbi.nlm.nih.gov/36921779/

82. PMID 36851997

Exosomal miR-320e as a Novel Potential Biomarker for Cerebral Small Vessel Disease.

Disposition: Included in a representative primary evidence table. Direct disease/model relevance or explicitly identified acute-stroke assay context.

Source: https://pubmed.ncbi.nlm.nih.gov/36851997/

83. PMID 36705010

Exosomes for the diagnosis and treatment of dementia.

Disposition: Contextual or additional relevant record. Relevant to mechanisms, vascular phenotype, methods or related literature; not all such records are cited or tabled in this narrative selection.

Source: https://pubmed.ncbi.nlm.nih.gov/36705010/

84. PMID 36646051

Evaluation of Microvascular Rarefaction in Vascular Cognitive Impairment and Heart Failure (CRUCIAL): Study Protocol for an Observational Study.

Disposition: Contextual or additional relevant record. Relevant to mechanisms, vascular phenotype, methods or related literature; not all such records are cited or tabled in this narrative selection.

Source: https://pubmed.ncbi.nlm.nih.gov/36646051/

85. PMID 36330621

Tandem Mass Tag (TMT) Quantitative Proteomic Analysis of Serum Exosomes in Cerebral Small-vessel Disease (CSVD) Patients With Depressive Symptoms.

Disposition: Contextual or additional relevant record. Relevant to mechanisms, vascular phenotype, methods or related literature; not all such records are cited or tabled in this narrative selection.

Source: https://pubmed.ncbi.nlm.nih.gov/36330621/

86. PMID 36232513

Mesenchymal Stem Cell-Derived Extracellular Vesicles as Proposed Therapy in a Rat Model of Cerebral Small Vessel Disease.

Disposition: Included in a representative primary evidence table. Direct disease/model relevance or explicitly identified acute-stroke assay context.

Source: https://pubmed.ncbi.nlm.nih.gov/36232513/

87. PMID 35966195

Circulating exo-miR-154-5p regulates vascular dementia through endothelial progenitor cell-mediated angiogenesis.

Disposition: Included in a representative primary evidence table. Direct disease/model relevance or explicitly identified acute-stroke assay context.

Source: https://pubmed.ncbi.nlm.nih.gov/35966195/

88. PMID 35841005

Exosomal miR-132-3p from mesenchymal stromal cells improves synaptic dysfunction and cognitive decline in vascular dementia.

Disposition: Included in a representative primary evidence table. Direct disease/model relevance or explicitly identified acute-stroke assay context.

Source: https://pubmed.ncbi.nlm.nih.gov/35841005/

89. PMID 35752392

MicroRNA-29c-3p in dual-labeled exosome is a potential diagnostic marker of subjective cognitive decline.

Disposition: Outside the primary narrative evidence scope. Primarily AD diagnostic development, not a direct VCID validation dataset; vascular-comparator relevance can be revisited in a broader review.

Source: https://pubmed.ncbi.nlm.nih.gov/35752392/

90. PMID 35547763

M2 microglia-derived extracellular vesicles promote white matter repair and functional recovery via miR-23a-5p after cerebral ischemia in mice.

Disposition: Contextual or additional relevant record. Relevant to mechanisms, vascular phenotype, methods or related literature; not all such records are cited or tabled in this narrative selection.

Source: https://pubmed.ncbi.nlm.nih.gov/35547763/

91. PMID 35470961

Amyloid-β protein and MicroRNA-384 in NCAM-Labeled exosomes from peripheral blood are potential diagnostic markers for Alzheimer's disease.

Disposition: Outside the primary narrative evidence scope. Primarily AD diagnostic development, not a direct VCID validation dataset; vascular-comparator relevance can be revisited in a broader review.

Source: https://pubmed.ncbi.nlm.nih.gov/35470961/

92. PMID 35368870

Neurofilament Light Chain (NF-L) Stimulates Lipid Peroxidation to Neuronal Membrane through Microglia-Derived Ferritin Heavy Chain (FTH) Secretion.

Disposition: Contextual or additional relevant record. Relevant to mechanisms, vascular phenotype, methods or related literature; not all such records are cited or tabled in this narrative selection.

Source: https://pubmed.ncbi.nlm.nih.gov/35368870/

93. PMID 35346374

AQP4 labels a subpopulation of white matter-dependent glial radial cells affected by pediatric hydrocephalus, and its expression increased in glial microvesicles released to the cerebrospinal fluid in obstructive hydrocephalus.

Disposition: Outside the primary narrative evidence scope. Neonatal/perinatal or pediatric injury differs from adult VCID.

Source: https://pubmed.ncbi.nlm.nih.gov/35346374/

94. PMID 35071475

Exosomal miRNA-223-3p as potential biomarkers in patients with cerebral small vessel disease cognitive impairment.

Disposition: Included in a representative primary evidence table. Direct disease/model relevance or explicitly identified acute-stroke assay context.

Source: https://pubmed.ncbi.nlm.nih.gov/35071475/

95. PMID 34650682

HNSC exosome-derived MIAT improves cognitive disorders in rats with vascular dementia via the miR-34b-5p/CALB1 axis.

Disposition: Included in a representative primary evidence table. Direct disease/model relevance or explicitly identified acute-stroke assay context.

Source: https://pubmed.ncbi.nlm.nih.gov/34650682/

96. PMID 34636491

The promise of exosome applications in treating central nervous system diseases.

Disposition: Contextual or additional relevant record. Relevant to mechanisms, vascular phenotype, methods or related literature; not all such records are cited or tabled in this narrative selection.

Source: https://pubmed.ncbi.nlm.nih.gov/34636491/

97. PMID 34312743

Exosomes derived from astrocytes after oxygen-glucose deprivation promote differentiation and migration of oligodendrocyte precursor cells in vitro.

Disposition: Contextual or additional relevant record. Relevant to mechanisms, vascular phenotype, methods or related literature; not all such records are cited or tabled in this narrative selection.

Source: https://pubmed.ncbi.nlm.nih.gov/34312743/

98. PMID 34176095

COVID-19 Infection and Circulating Microparticles-Reviewing Evidence as Microthrombogenic Risk Factor for Cerebral Small Vessel Disease.

Disposition: Contextual or additional relevant record. Relevant to mechanisms, vascular phenotype, methods or related literature; not all such records are cited or tabled in this narrative selection.

Source: https://pubmed.ncbi.nlm.nih.gov/34176095/

99. PMID 34024835

Altered Amyloid-β and Tau Proteins in Neural-Derived Plasma Exosomes of Type 2 Diabetes Patients with Orthostatic Hypotension.

Disposition: Contextual or additional relevant record. Relevant to mechanisms, vascular phenotype, methods or related literature; not all such records are cited or tabled in this narrative selection.

Source: https://pubmed.ncbi.nlm.nih.gov/34024835/

100. PMID 33869172

Human Wharton's Jelly Mesenchymal Stromal Cell-Derived Small Extracellular Vesicles Drive Oligodendroglial Maturation by Restraining MAPK/ERK and Notch Signaling Pathways.

Disposition: Outside the primary narrative evidence scope. Neonatal/perinatal or pediatric injury differs from adult VCID.

Source: https://pubmed.ncbi.nlm.nih.gov/33869172/

101. PMID 33749657

Changes in the Morphology, Number, and Protein Levels of Plasma Exosomes in CADASIL Patients.

Disposition: Contextual or additional relevant record. Relevant to mechanisms, vascular phenotype, methods or related literature; not all such records are cited or tabled in this narrative selection.

Source: https://pubmed.ncbi.nlm.nih.gov/33749657/

102. PMID 33718454

Diets and Cellular-Derived Microparticles: Weighing a Plausible Link With Cerebral Small Vessel Disease.

Disposition: Contextual or additional relevant record. Relevant to mechanisms, vascular phenotype, methods or related literature; not all such records are cited or tabled in this narrative selection.

Source: https://pubmed.ncbi.nlm.nih.gov/33718454/

103. PMID 33498429

Elevated Circulating Microparticle Subpopulations in Incidental Cerebral White Matter Hyperintensities: A Multimodal Study.

Disposition: Contextual or additional relevant record. Relevant to mechanisms, vascular phenotype, methods or related literature; not all such records are cited or tabled in this narrative selection.

Source: https://pubmed.ncbi.nlm.nih.gov/33498429/

104. PMID 33235449

In vivo Monitoring and Assessment of Exogenous Mesenchymal Stem Cell-Derived Exosomes in Mice with Ischemic Stroke by Molecular Imaging.

Disposition: Contextual or additional relevant record. Relevant to mechanisms, vascular phenotype, methods or related literature; not all such records are cited or tabled in this narrative selection.

Source: https://pubmed.ncbi.nlm.nih.gov/33235449/

105. PMID 32889008

Exosomes derived from bone marrow mesenchymal stem cells harvested from type two diabetes rats promotes neurorestorative effects after stroke in type two diabetes rats.

Disposition: Contextual or additional relevant record. Relevant to mechanisms, vascular phenotype, methods or related literature; not all such records are cited or tabled in this narrative selection.

Source: https://pubmed.ncbi.nlm.nih.gov/32889008/

106. PMID 32440476

ESC-sEVs Rejuvenate Senescent Hippocampal NSCs by Activating Lysosomes to Improve Cognitive Dysfunction in Vascular Dementia.

Disposition: Included in a representative primary evidence table. Direct disease/model relevance or explicitly identified acute-stroke assay context.

Source: https://pubmed.ncbi.nlm.nih.gov/32440476/

107. PMID 32410865

Role of Mitochondrial Stress Protein HSP60 in Diabetes-Induced Neuroinflammation.

Disposition: Outside the primary narrative evidence scope. Different condition or outcome without direct VCID/white-matter EV evidence for the primary map.

Source: https://pubmed.ncbi.nlm.nih.gov/32410865/

108. PMID 32384937

Alzheimer's disease progression characterized by alterations in the molecular profiles and biogenesis of brain extracellular vesicles.

Disposition: Outside the primary narrative evidence scope. Different condition or outcome without direct VCID/white-matter EV evidence for the primary map.

Source: https://pubmed.ncbi.nlm.nih.gov/32384937/

109. PMID 32248519

Extracellular vesicles containing the transferrin receptor as nanocarriers of apotransferrin.

Disposition: Contextual or additional relevant record. Relevant to mechanisms, vascular phenotype, methods or related literature; not all such records are cited or tabled in this narrative selection.

Source: https://pubmed.ncbi.nlm.nih.gov/32248519/

110. PMID 32184939

C-C chemokine receptor type 2-overexpressing exosomes alleviated experimental post-stroke cognitive impairment by enhancing microglia/macrophage M2 polarization.

Disposition: Included in a representative primary evidence table. Direct disease/model relevance or explicitly identified acute-stroke assay context.

Source: https://pubmed.ncbi.nlm.nih.gov/32184939/

111. PMID 32135232

Profile of Pathogenic Proteins and MicroRNAs in Plasma-derived Extracellular Vesicles in Alzheimer's Disease: A Pilot Study.

Disposition: Contextual or additional relevant record. Relevant to mechanisms, vascular phenotype, methods or related literature; not all such records are cited or tabled in this narrative selection.

Source: https://pubmed.ncbi.nlm.nih.gov/32135232/

112. PMID 32075692

Low dose of extracellular vesicles identified that promote recovery after ischemic stroke.

Disposition: Contextual or additional relevant record. Relevant to mechanisms, vascular phenotype, methods or related literature; not all such records are cited or tabled in this narrative selection.

Source: https://pubmed.ncbi.nlm.nih.gov/32075692/

113. PMID 31993045

Brain-Derived Microparticles (BDMPs) Contribute to Neuroinflammation and Lactadherin Reduces BDMP Induced Neuroinflammation and Improves Outcome After Stroke.

Disposition: Contextual or additional relevant record. Relevant to mechanisms, vascular phenotype, methods or related literature; not all such records are cited or tabled in this narrative selection.

Source: https://pubmed.ncbi.nlm.nih.gov/31993045/

114. PMID 31931927

Effects of Butyphthalide Combined with Idebenone on Inflammatory Cytokines and Vascular Endothelial Functions of Patients with Vascular Dementia.

Disposition: Outside the primary narrative evidence scope. Non-EV analyte, drug-only outcome, or an assay reagent termed microparticle rather than EV biology.

Source: https://pubmed.ncbi.nlm.nih.gov/31931927/

115. PMID 31879236

Plasma biomarkers of astrocytic and neuronal dysfunction in early- and late-onset Alzheimer's disease.

Disposition: Outside the primary narrative evidence scope. Different condition or outcome without direct VCID/white-matter EV evidence for the primary map.

Source: https://pubmed.ncbi.nlm.nih.gov/31879236/

116. PMID 31708792

A Novel Model of Mixed Vascular Dementia Incorporating Hypertension in a Rat Model of Alzheimer's Disease.

Disposition: Contextual or additional relevant record. Relevant to mechanisms, vascular phenotype, methods or related literature; not all such records are cited or tabled in this narrative selection.

Source: https://pubmed.ncbi.nlm.nih.gov/31708792/

117. PMID 31691877

Specific Signatures of Serum miRNAs as Potential Biomarkers to Discriminate Clinically Similar Neurodegenerative and Vascular-Related Diseases.

Disposition: Outside the primary narrative evidence scope. Non-EV analyte, drug-only outcome, or an assay reagent termed microparticle rather than EV biology.

Source: https://pubmed.ncbi.nlm.nih.gov/31691877/

118. PMID 31683489

Flotillin is a Novel Diagnostic Blood Marker of Alzheimer's Disease.

Disposition: Outside the primary narrative evidence scope. Non-EV analyte, drug-only outcome, or an assay reagent termed microparticle rather than EV biology.

Source: https://pubmed.ncbi.nlm.nih.gov/31683489/

119. PMID 31398924

Intranasally Administered Exosomes from Umbilical Cord Stem Cells Have Preventive Neuroprotective Effects and Contribute to Functional Recovery after Perinatal Brain Injury.

Disposition: Outside the primary narrative evidence scope. Neonatal/perinatal or pediatric injury differs from adult VCID.

Source: https://pubmed.ncbi.nlm.nih.gov/31398924/

120. PMID 31394992

MiR-126 Mediates Brain Endothelial Cell Exosome Treatment-Induced Neurorestorative Effects After Stroke in Type 2 Diabetes Mellitus Mice.

Disposition: Contextual or additional relevant record. Relevant to mechanisms, vascular phenotype, methods or related literature; not all such records are cited or tabled in this narrative selection.

Source: https://pubmed.ncbi.nlm.nih.gov/31394992/

121. PMID 31137000

CROI 2019: neurologic complications of HIV disease.

Disposition: Outside the primary narrative evidence scope. Different condition or outcome without direct VCID/white-matter EV evidence for the primary map.

Source: https://pubmed.ncbi.nlm.nih.gov/31137000/

122. PMID 30898154

Exosomes derived from umbilical cord mesenchymal stem cells reduce microglia-mediated neuroinflammation in perinatal brain injury.

Disposition: Outside the primary narrative evidence scope. Neonatal/perinatal or pediatric injury differs from adult VCID.

Source: https://pubmed.ncbi.nlm.nih.gov/30898154/

123. PMID 30629763

Brain-derived and circulating vesicle profiles indicate neurovascular unit dysfunction in early Alzheimer's disease.

Disposition: Contextual or additional relevant record. Relevant to mechanisms, vascular phenotype, methods or related literature; not all such records are cited or tabled in this narrative selection.

Source: https://pubmed.ncbi.nlm.nih.gov/30629763/

124. PMID 30617992

Altered Extracellular Vesicle MicroRNA Expression in Ischemic Stroke and Small Vessel Disease.

Disposition: Included in a representative primary evidence table. Direct disease/model relevance or explicitly identified acute-stroke assay context.

Source: https://pubmed.ncbi.nlm.nih.gov/30617992/

125. PMID 30483114

"Liquid Biopsy" of White Matter Hyperintensity in Functionally Normal Elders.

Disposition: Included in a representative primary evidence table. Direct disease/model relevance or explicitly identified acute-stroke assay context.

Source: https://pubmed.ncbi.nlm.nih.gov/30483114/

126. PMID 30170428

Plasma brain natriuretic peptide is a biomarker for screening ischemic cerebral small vessel disease in patients with hypertension.

Disposition: Outside the primary narrative evidence scope. Non-EV analyte, drug-only outcome, or an assay reagent termed microparticle rather than EV biology.

Source: https://pubmed.ncbi.nlm.nih.gov/30170428/

127. PMID 29650593

Human Neural Stem Cell Extracellular Vesicles Improve Recovery in a Porcine Model of Ischemic Stroke.

Disposition: Outside the primary narrative evidence scope. Broad background or indirect relevance; outside the representative primary evidence set.

Source: https://pubmed.ncbi.nlm.nih.gov/29650593/

128. PMID 29606187

The Serum Exosome Derived MicroRNA-135a, -193b, and -384 Were Potential Alzheimer's Disease Biomarkers.

Disposition: Outside the primary narrative evidence scope. Primarily AD diagnostic development, not a direct VCID validation dataset; vascular-comparator relevance can be revisited in a broader review.

Source: https://pubmed.ncbi.nlm.nih.gov/29606187/

129. PMID 27468268

Blood Biomarkers for Evaluation of Perinatal Encephalopathy.

Disposition: Outside the primary narrative evidence scope. Neonatal/perinatal or pediatric injury differs from adult VCID.

Source: https://pubmed.ncbi.nlm.nih.gov/27468268/

130. PMID 27350079

Cilostazol reduces blood brain barrier dysfunction, white matter lesion formation and motor deficits following chronic cerebral hypoperfusion.

Disposition: Contextual or additional relevant record. Relevant to mechanisms, vascular phenotype, methods or related literature; not all such records are cited or tabled in this narrative selection.

Source: https://pubmed.ncbi.nlm.nih.gov/27350079/

131. PMID 27142381

Assessment and Imaging of the Cerebrovascular Glycocalyx.

Disposition: Contextual or additional relevant record. Relevant to mechanisms, vascular phenotype, methods or related literature; not all such records are cited or tabled in this narrative selection.

Source: https://pubmed.ncbi.nlm.nih.gov/27142381/

132. PMID 24976400

Microparticles generated during chronic cerebral ischemia deliver proapoptotic signals to cultured endothelial cells.

Disposition: Contextual or additional relevant record. Relevant to mechanisms, vascular phenotype, methods or related literature; not all such records are cited or tabled in this narrative selection.

Source: https://pubmed.ncbi.nlm.nih.gov/24976400/

133. PMID 24029712

Circulating markers of endothelial dysfunction and platelet activation in patients with severe symptomatic cerebral small vessel disease.

Disposition: Contextual or additional relevant record. Relevant to mechanisms, vascular phenotype, methods or related literature; not all such records are cited or tabled in this narrative selection.

Source: https://pubmed.ncbi.nlm.nih.gov/24029712/

134. PMID 19723448

Potential roles of cell-derived microparticles in ischemic brain disease.

Disposition: Contextual or additional relevant record. Relevant to mechanisms, vascular phenotype, methods or related literature; not all such records are cited or tabled in this narrative selection.

Source: https://pubmed.ncbi.nlm.nih.gov/19723448/

135. PMID 19723444

Translation of stroke research into stroke therapy.

Disposition: Contextual or additional relevant record. Relevant to mechanisms, vascular phenotype, methods or related literature; not all such records are cited or tabled in this narrative selection.

Source: https://pubmed.ncbi.nlm.nih.gov/19723444/

136. PMID 17876771

Transendothelial migration of leukocytes is promoted by plasma from a subgroup of immune thrombocytopenic purpura patients with small-vessel ischemic brain disease.

Disposition: Contextual or additional relevant record. Relevant to mechanisms, vascular phenotype, methods or related literature; not all such records are cited or tabled in this narrative selection.

Source: https://pubmed.ncbi.nlm.nih.gov/17876771/

137. PMID 12565721

Vascular dementia in patients with immune thrombocytopenic purpura.

Disposition: Contextual or additional relevant record. Relevant to mechanisms, vascular phenotype, methods or related literature; not all such records are cited or tabled in this narrative selection.

Source: https://pubmed.ncbi.nlm.nih.gov/12565721/

138. PMID 12430912

Idiopathic thrombocytopenic purpura: pathophysiology and management.

Disposition: Contextual or additional relevant record. Relevant to mechanisms, vascular phenotype, methods or related literature; not all such records are cited or tabled in this narrative selection.

Source: https://pubmed.ncbi.nlm.nih.gov/12430912/

139. PMID 7583683

Cerebrospinal fluid 'neuronal thread protein' comes from serum by passage over the blood-brain barrier.

Disposition: Outside the primary narrative evidence scope. Non-EV analyte, drug-only outcome, or an assay reagent termed microparticle rather than EV biology.

Source: https://pubmed.ncbi.nlm.nih.gov/7583683/

140. PMID 8303669

Elevated platelet microparticles in transient ischemic attacks, lacunar infarcts, and multiinfarct dementias.

Disposition: Contextual or additional relevant record. Relevant to mechanisms, vascular phenotype, methods or related literature; not all such records are cited or tabled in this narrative selection.

Source: https://pubmed.ncbi.nlm.nih.gov/8303669/

Citation-traced primary study

Buntsma et al. (reference 43), DOI 10.1007/s12975-025-01371-z. Included as acute-stroke assay context in Table 5; retrieved by source tracing, not in the 140-record expanded query. The PubMed retrieval count therefore must not be treated as the complete table-selection denominator.

Targeted correction and full-text checks

The corrigendum to Wang et al. (3), DOI 10.1016/j.expneurol.2026.115967 (reference 65), was checked in a targeted source review on 9 October 2026 together with the supplied primary full texts. It is not counted as an additional efficacy study or as a record returned by the 140-record PubMed query. The correction replaces representative Golgi/DHE images and a beta-actin band; it does not supply revised Methods or a statement that conclusions are unchanged. The complete main article for Feng et al. (46) was checked on 10 October 2026. This subsequent method extraction updates Table 5 and S2 without changing the 8 October search yield. We checked Supporting Table S1 for Wang et al. (6), supplied as syn22268-sup-0001-tables1.docx, on 10 October 2026. The five starting denominators are 12 each; reported seven-day deaths are 1, 4, 2, 2 and 4 in Sham, VaD, EV, EV plus vehicle and EV plus LY294002 groups. This attachment check does not change the database search yield.
