# Supplementary material for: Extracellular vesicles in vascular cognitive impairment and dementia: a neurovascular evidence map for patient stratification and clinical translation
Source: Front Neurol. 2026 Oct 30;17:1986081. doi: 10.3389/fneur.2026.1986081 (PMC13760277; doi:10.3389/fneur.2026.1986081)
Supplement: Supplementary file 2 [file Data_Sheet_2.PDF]

# Supplementary search and methodological appraisal

Manuscript 1986081 | Frontiers in Neurology

## Supplementary Material S1

The expanded revision-stage search was run on 8 October 2026 and returned 140 PubMed records. The complete query and returned records, with narrative disposition notes, accompany this document in the S1 search-record file. The original July 2026 export and screening log were not retained and are not reconstructed.

### Executable PubMed query

("Extracellular Vesicles"[MeSH] OR "extracellular vesicle*"[tiab] OR exosome*[tiab] OR microvesicle*[tiab] OR microparticle*[tiab] OR ectosome*[tiab] OR "small EV*"[tiab] OR "extracellular particle*"[tiab]) AND ("Dementia, Vascular"[MeSH] OR "vascular cognitive impairment"[tiab] OR "vascular cognitive impairment and dementia"[tiab] OR "vascular dementia"[tiab] OR "cerebral small vessel disease"[tiab] OR "small vessel disease"[tiab] OR "chronic cerebral hypoperfusion"[tiab] OR "cerebral hypoperfusion"[tiab] OR "post-stroke cognitive impairment"[tiab] OR "poststroke cognitive impairment"[tiab] OR ("white matter"[tiab] AND (ischemi*[tiab] OR ischaemi*[tiab] OR hypoperfus*[tiab]))) AND ("1900/01/01"[Date - Publication] : "2026/10/08"[Date - Publication])

This search improves terminology coverage but is PubMed-led and narrative. Titles/abstracts and representative source details were extracted with AI assistance; independent duplicate screening and formal risk-of-bias scoring were not performed. Tabled primary studies, contextual candidates, out-of-scope records and correction notices are distinguished in the accompanying record. Buntsma et al. (43) was retrieved by citation tracing and is not in the expanded-query return set.

## Supplementary Material S2

Methodological appraisal is descriptive and use-specific. The items below apply MISEV2023 to reported features without awarding compliance points, assigning readiness levels, treating historical studies as violations of later guidance, or interpreting unlisted methods as confirmed omissions.

### Preparation and nomenclature

Record matrix, source-cell conditions, separation/enrichment and measured size distribution. Use EV for mixed or incompletely size-defined preparations. Reserve exosome for supported endosomal origin. Older microparticle evidence is contextual, not equivalent to sEV products.

### Particle measurement

Record detection method and thresholds, concentration/dose units and calibration. NTA or particle counters can detect non-EV nanoparticles. Protein mass, particle number and parent-cell equivalents are not interchangeable dose units.

### EV characterization

Use complementary physical and biochemical observations. Membrane and cytosolic EV-associated markers support identity. Selected cellular negative markers do not establish complete purity.

### Purity and non-EV controls

Assess matrix-appropriate lipoproteins, albumin, soluble proteins, RNA/protein complexes and cellular contaminants. Orthogonal separation, process-matched controls and EV-depleted fractions help attribute assay signals or effects.

### Cellular origin

Distinguish source-cell production in culture, operational immunoenrichment and demonstrated cellular origin in biofluids. Marker specificity, intact-particle colocalization, soluble-marker controls and additional phenotyping matter. CD31 alone is not brain-endothelial-specific.

### Functional attribution

Cargo gain/loss or rescue supports a mechanism but should be interpreted with preparation controls and off-target effects. Lipid dyes or reporter transfer alone do not prove intact EV transport and functional cargo delivery.

### Human validation

Record cohort sizes and overlaps, discovery versus verification/validation, covariates, longitudinal selection, locked model evaluation, calibration and prospective clinical decisions. Separate datasets within a programme are distinct from independent laboratory replication or clinical utility.

## Study-specific extraction and interpretation

### Hu et al. (15)

Differential ultracentrifugation, TEM, nano-flow size/concentration and CD9/CD63/TSG101 with GM130, beta-actin and Lamin A/C controls were reported. These selected negative controls do not establish all soluble or non-vesicular components absent. Source https://pmc.ncbi.nlm.nih.gov/articles/PMC7237844/

### Ma et al. (16)

Particle-size/morphology measurements, CD63 and MSC-associated CD29 were reported, with particle-based dosing. These observations support the described preparation but do not establish endosomal biogenesis or all-matrix purity. Source https://pmc.ncbi.nlm.nih.gov/articles/PMC9284820/

### Guy et al. (47)

Differential centrifugation/ultracentrifugation, ultrafiltration, NTA, TEM and EV/MSC surface-marker profiling were reported. Comparability still requires common yield, dose and potency units. Source https://pmc.ncbi.nlm.nih.gov/articles/PMC9569832/

### Yang et al. (5)

Microglial culture supernatants after sinomenine exposure were processed with an Umibio extraction/purification kit. TEM, NTA and CD63/CD81 were reported. Process-matched, residual-drug and non-EV controls are relevant to functional attribution. Source https://pmc.ncbi.nlm.nih.gov/articles/PMC11881310/

### Pan et al. (4)

Cultured NSC preparations used differential ultracentrifugation; NTA, TEM and CD63/TSG101 were reported. Hippocampal Nestin-associated capture is a separate source-attribution procedure and should not be conflated with cultured NSC-EV identity. A correction dated 18 July 2026 concerns Fig. 5E. Source https://pmc.ncbi.nlm.nih.gov/articles/PMC12239277/ and correction DOI 10.1186/s40478-026-02358-z.

### Shen et al. (37)

hUCMSC supernatants underwent differential centrifugation, filtration and ultracentrifugation before cNGR conjugation. NTA/TEM and EV markers with calnexin/GM130 were reported. A sucrose cushion was used to remove free labeling dye. These controls support technical characterization without establishing a clinical release specification. Source https://pmc.ncbi.nlm.nih.gov/articles/PMC13240518/

### Zheng et al. (40)

Bacterial culture supernatants underwent sequential centrifugation, 0.45/0.22-micrometre filtration, 100 kDa concentration and 150,000g pelleting, followed by PBS washes and metabolite loading. This bacterial

product needs its own identity, purity and microbiological controls. Source https://pmc.ncbi.nlm.nih.gov/articles/PMC13343794/

**Lin et al. (56)**

Differential ultracentrifugation and TEM/NTA/WB were reported. Muscle-specific CD63-EGFP tracing and miRNA/pathway perturbations strengthen source-function attribution, although tissue dissociation and labeling remain relevant technical controls. Source https://pmc.ncbi.nlm.nih.gov/articles/PMC12279231/

**Qi et al. (57)**

Neural stem cell conditioned medium underwent ultracentrifugation; morphology/particle characterization and MIAT-pathway perturbation were reported. Biological activity does not establish standardized product purity or long-term safety. Source https://pmc.ncbi.nlm.nih.gov/articles/PMC8507051/

**Yang et al. (59)**

Differential ultracentrifugation, TEM, qNano and CD9/CD63/CD81 characterization were reported. CCR2 expression, CCL2 binding and exosome-free supernatant comparison support the proposed mechanism. Source https://pmc.ncbi.nlm.nih.gov/articles/PMC7062036/

**Elahi et al. (51)**

Plasma precipitation followed sequential CD31/CD146 immunoenrichment. Cargo ELISAs were normalized to CD81. Particle-size support referred to earlier work using the same preparation approach; this is not new size characterization of every sample in this cohort. The source explicitly recognizes residual preparation uncertainty. Source https://pmc.ncbi.nlm.nih.gov/articles/PMC6244607/

**van Kralingen et al. (52)**

Serum precipitation, TEM/NTA and microarray/qPCR were reported. Discovery/validation cohorts were 39/173. The reported SVD subtype signal does not establish chronic VCID diagnosis or prognosis. Source https://pmc.ncbi.nlm.nih.gov/articles/PMC6733813/

**Zhao et al. (44)**

Umibio kit enrichment, TEM/NTA and RT-PCR with an external miRNA reference were reported. Table 1 and abstract give 40/38/35 participants, while narrative results contain inconsistent counts. Statistical methods describe comparison, correlation and ROC analyses, not an adjusted cognitive-outcome prediction model. Source https://atm.amegroups.org/article/view/85896/html

**Han et al. (22)**

Serum was filtered and ultracentrifuged; TEM/NTA and sequencing/RT-qPCR were reported. Discovery used seven VaD and seven age-matched controls. The source reports subsequent clinical verification n=23 and a larger recruited sample with differently aged controls; these stages should not be called external prospective validation. Source https://www.frontiersin.org/journals/cellular-neuroscience/articles/10.3389/fncel.2022.881175/full

**Gao et al. (45)**

The 150-case/80-control study used a plasma precipitation reagent with proteinase K processing, physical characterization and EV-associated marker analysis. Microarray discovery pooled samples from 16 cases/16 controls. The WMH regression adjusts for selected covariates but does not validate cognitive prognosis. Source https://pmc.ncbi.nlm.nih.gov/articles/PMC9961587/

**Qi et al. (53)**

Plasma TiO2 microsphere affinity enrichment, TEM, NTA/DLS and CD63/TSG101/CD81/CD9 western blotting were reported. The 10/10 proteomic comparison is exploratory. Co-isolated protein and lipoprotein contributions need direct controls rather than inference from morphology. Source https://pmc.ncbi.nlm.nih.gov/articles/PMC10258006/

**Yang et al. (38)**

EDTA plasma and an antibody-affinity bead platform were used, with TEM, particle characterization, TSG101/CD9/CD81 and calnexin. LC-MS/MS discovery was followed by PRM. The article reports n=100 and n=211, but its validation subgroup counts (55+45+76+38) sum to 214 and its introductory AD count differs. Models include demographics/vascular/imaging variables and internal calibration. Source https://link.springer.com/article/10.1186/s13195-026-02078-5

**You et al. (39)**

Publisher supplementary tables specify discovery n=55, validation n=291 and followed n=87. Public extended data describe CD31/GLUT1 nano-flow cross-validation and NTA comparison. Supplementary Table 8 lists demographic, vascular-risk and AD-biomarker adjustment. Supplementary Table 6 reports validation VCI-vs-AD AUC 0.762 for c-BEEVs and 0.965 with p-tau181. Supplementary Table 3 shows baseline differences between followed/non-followed participants. CD31/GLUT1 results support, but do not make infallible, cellular attribution. Source https://www.nature.com/articles/s43587-026-01117-y and publisher Supplementary Information.

**Buntsma et al. (43)**

EDTA plasma was double centrifuged. EV concentrations were measured by immunostaining and calibrated flow cytometry using a reported 100-1000 nm range, fluorescence/size calibration, isotype controls and MIFlowCyt-EV reporting. This is a broader EV detection window, not an isolated small-EV preparation. Source https://link.springer.com/article/10.1007/s12975-025-01371-z

**Wang et al. (3) and corrigendum (65)**

Embryonic mouse NSC conditioned medium underwent serial centrifugation and 100,000g ultracentrifugation for 90 min. NTA, TEM and CD63/TSG101 were reported. The reported size was approximately 100 +/- 55 nm. miR-132-3p gain/loss preparations, wild-type/mutant RASA1 reporter testing and RASA1 overexpression in recipient neurons support a pathway contribution. Intranasal treatment began 24 h after surgery, with 1 x 10^10 particles weekly for three weeks. The cognitive improvement reported for the enriched preparation should not be generalized to every unmodified preparation or behavioural comparison. Hippocampal Nestin-associated capture is an operational enrichment step; PKH26 colocalization does not prove intact-vesicle cargo delivery. The main article uses hypoxia/reoxygenation (H/R) in Methods but oxygen-glucose deprivation (OGD) in Results and contains inconsistent wording for some mechanism directions and sample counts. We therefore retain only clearly supported preparation and pathway descriptions. Purity and functional-attribution controls remain incompletely specified in the main article; the separate supplementary material was not available for review. The 2026 corrigendum replaces Fig. 2F enriched-group Golgi imagery, Fig. 3A depleted-group DHE imagery and the Fig. 5C beta-actin band. It provides no revised Methods or statement that the conclusions are unchanged. Sources https://doi.org/10.1016/j.expneurol.2025.115224 and https://doi.org/10.1016/j.expneurol.2026.115967.

**Wang et al. (6)**

hUCMSCs were washed and transferred from FBS-containing medium to serum-free collection medium for 48 h. The main article describes an SBI extraction kit together with ultracentrifugation, without complete high-speed separation parameters. TEM, NTA (46-150 nm, peak near 100 nm), CD63/HSPA8/TSG101 and a calnexin control were reported. Bilateral carotid-ligation rats received tail-vein treatment from the next day for seven days; the dose is reported as 100 ng/kg/day and is not converted to a different unit. We checked Supporting Table S1 on 10 October 2026. It reports seven-day deaths of 1/12 (Sham), 4/12 (VaD), 2/12 (EVs), 2/12 (EVs plus vehicle) and 4/12 (EVs plus LY294002). These denominators agree with the 60 starting rats. The main article reports eight animals per endpoint group but does not explain selection from groups with more than eight survivors. The table provides no between-group mortality test; the counts do not establish a survival benefit. LY294002 attenuated pathway, microglial-marker and protective effects, supporting PI3K-sensitive activity. No LY294002-only group, Nrf2-specific rescue or active-cargo test establishes an EV-specific source-cargo-recipient mechanism. GW4869 appears in preparation characterization, not as an animal functional EV-depletion control. Calnexin and serum-free collection do

not exclude all non-EV material or establish batch potency. Source https://doi.org/10.1002/syn.22268 and Supporting Table S1.

### Liu et al. (58)

Human induced pluripotent stem cell (iPSC)-derived MSC conditioned medium underwent differential centrifugation, 0.22-micrometre filtration and 100,000g pelleting for 70 min. TEM, calibrated NanoFCM (60-120 nm), CD9/CD63/TSG101 and GM130 were reported. NanoFCM is not NTA. After MCAO, tail-vein treatment began at 4 h with 1 x 10^11 particles in 500 microlitres and was repeated every three days until assessment. The 90-day Y-maze used n=10 mice per group; tissue and cell endpoints generally report n=3 replicates. Thus, a late assessment does not demonstrate reversal following delayed treatment of established cognitive impairment. NSC senescence-associated and neurogenic readouts improved, but the authors acknowledge that NSC rejuvenation is not proven to mediate the synaptic or cognitive benefit. Functional cargo was not identified; miR-17-92 and miR-133b in the Discussion refer to prior work. GM130 is a selected cellular-contamination control, not proof that all soluble or non-EV material is absent. Source https://doi.org/10.1007/s12031-024-02191-w.

### Feng et al. (46)

The complete main article was checked on 10 October 2026. It compares 18 participants with CSVD-related cognitive impairment and 18 healthy controls. Blood was collected in heparin-anticoagulant tubes and centrifuged at 2000g for 15 min at 4 degrees C. The collection paragraph then calls the material serum, whereas the isolation Methods use plasma. The reported anticoagulation workflow supports a plasma interpretation; the internal discrepancy remains unresolved. After thawing, 3000g/10000g centrifugation and 0.22-micrometre filtration preceded PTM-EVS kit enrichment. TEM and NTA were reported, with a 112-nm particle-size peak. Proteomic detection of PDCD6IP does not replace independent positive/negative marker validation or matrix-contaminant assessment, which were not described in the main article. TMT-10PLEX identified 30 differential proteins (22 downregulated and 8 upregulated). Results report 781 quantified proteins, whereas Fig. 1c labels 781 as unique and 659 as quantified; these totals are not reconciled by inference. Donor pooling, channel allocation and biological/technical replicate design were not specified. The 60 fractions combined into 14 refer to peptide fractionation, not demonstrated donor pooling. The 1% identification FDR does not establish multiplicity control for differential-expression or enrichment testing. Baseline comparisons do not replace clinical covariate adjustment. Control mean MMSE (26.72) is below the stated impairment threshold of 27, leaving uncertainty about group definitions. No ROC analysis, covariate-adjusted clinical model or independent validation cohort was described. Data are available on request; the main article does not identify a separate supplementary methods file. Source https://doi.org/10.1016/j.neulet.2024.137631.

### Remaining extraction boundaries

We have now checked the complete primary articles for references 3, 6, 46 and 58 and Supporting Table S1 for reference 6. The separate supplementary file for reference 3 remains unavailable. Unspecified methods or unresolved inconsistencies are distinguished from unavailable full articles, and an unlisted detail is not evidence that the investigators omitted the procedure. No retrospective numerical MISEV compliance verdict is assigned.

## Source-to-domain provenance

1 Biological rationale. MISEV attribution/function principles (25); mechanisms in Table 3

2 Technical control. MISEV2023 (25); assay/product development (17-20,41)

3 Phenotype-relevant translation. VCID biomarker/trial guidance (21,31,32)

4 Clinical validation. Clinical biomarker and product development (19,21,31,32,41)

5 Prospective evaluation. Trial/utility preparation principles (21,32,41)
